# Generating Population-Linked Case Vignettes From Nationwide Triage Data: Framework Development and Validation Study

**DOI:** 10.2196/104801

**Published:** 2026-11-03

**Authors:** Anja Seidel, Edgar Steiger, Josephine Schuster, Lars Eric Kroll

**Affiliations:** 1 Department of IT and Data Science, Central Research Institute of Ambulatory Health Care (Zi), Salzufer 8, Berlin, 10587, Germany, 49 30 220056186; 2 Department of Medicine, Central Research Institute of Ambulatory Health Care (Zi), Berlin, Germany

**Keywords:** data science, medical informatics, clinical decision support systems, triage, machine learning, clustering algorithms, natural language processing, large language models

## Abstract

**Background:**

Digital decision-support tools such as triage systems and symptom checkers support millions of health-related decisions each year. Their quality and safety are commonly evaluated using textual patient cases, known as case vignettes. However, expert-written vignette sets cover only a limited spectrum of real-world patient presentations and lack population weights, which would allow extrapolating evaluation results to the underlying patient population.

**Objective:**

This study aimed to develop a data-driven framework for automatically generating a human-manageable set of case vignettes from nationwide triage data that capture broad presentation diversity and link each vignette to a quantitative weight reflecting the number of underlying patient assessments.

**Methods:**

From 3.2 million triage assessments conducted over 1 year using structured triage software in the German medical on-call service (telephone triage and online self-triage) and at joint contact points of the outpatient emergency care service and hospital emergency departments, we randomly sampled 50,000 cases. Triage questionnaires were converted into semantic embeddings using a German Sentence Transformer model and grouped by agglomerative clustering. For clusters containing sufficient assessments, we generated one representative assessment using a 2-phase simulated annealing optimization that minimized the distance from the cluster centroid while maximizing the number of answered triage questions. Each representative assessment was assigned the size of its source cluster as its sample-based weight. A similarity-based sensitivity analysis assessed whether these weights were preserved in the full 1-year population. Finally, question-answer pairs of the representative assessments were converted into textual case vignettes using controlled prompting of a large language model.

**Results:**

The cluster analysis yielded 514 included clusters covering 96.8% of the sampled 50,000 assessments. The generated representatives showed strong agreement with the majority treatment urgency recommendation of their source cluster (Spearman ρ=0.78; *P*<.001) and contained, on average, 4.3 more answered triage questions than the original assessments within their clusters. When weighted by cluster size, the representatives approximated the sample distributions of treatment urgency, demographics, and symptoms, although some systematic deviations remained, most notably an overrepresentation of female cases (13.5%), patients aged 14‐49 (8.0%) years, and the urgency category “As soon as possible” (6.6%). Of 121 recorded symptoms, 101 (83.5%) were covered by the representatives; the rest each occurred in <0.5% of the sample. Cluster-based vignette weights were strongly correlated with similarity-based population weights (Spearman ρ=0.77; *P*<.001), and 90.1% of assessments in the full 1-year population matched at least one vignette.

**Conclusions:**

We present a data-driven framework for deriving a manageable set of population-weighted case vignettes from nationwide triage data. The resulting vignettes captured broad presentation diversity, approximated key sample characteristics, and provided an explicit quantitative link to the number of underlying assessments. After medical expert review and refinement, the vignettes may support more population-aware evaluation and quality assurance of digital decision-support tools.

## Introduction

### Background

Digital decision-support tools, such as triage systems and symptom checkers, are increasingly used by both health care professionals and medical laypeople [1-3]. Beyond stand-alone consumer applications, triage systems are also embedded in routine health care services, including nationwide access services, where they support treatment decisions in emergency medical services and medical on-call services. Comparable nationwide health care access services rely on triage systems, including 116117 in Germany [3], NHS 111 in the United Kingdom [4], 1177 in Sweden [5], and Healthdirect in Australia [6]. In these settings, triage not only classifies urgency but also helps direct patients to appropriate points of care. Rather than investigating longitudinal clinical pathways across multiple care settings, this study focuses on the first step in the care process: the patient presentation and standardized triage assessment recorded at first contact with the German medical on-call service (116117).

The widespread use of these tools, together with the large number of patients affected, underscores the importance of ongoing quality assurance. In addition, given that an increasing number of alternative products are available that, despite conforming to medical device regulations, appear to achieve different and sometimes conflicting results (eg, [7,8]), national agencies need to make informed decisions concerning the choice of triage software. In triage settings, accurate identification of urgent cases is essential to ensure timely referral to appropriate medical care, whereas less urgent cases should be de-escalated accordingly. Errors in either direction can have major consequences. Undertriage, in which a serious case is classified as not urgent enough, can lead to health- or even life-threatening delays, whereas overtriage, in which patients are assigned an unnecessarily high level of urgency, consumes limited health care resources and may delay treatment of truly urgent patients.

Triage tools have been evaluated using both real patient encounters (eg, [9,10]) and standardized medical case vignettes (eg, [5,11]), that is, textual descriptions of patient presentations. Evaluations based on real patient encounters can reflect routine-use conditions, but they are often restricted to the specific cases observed during a given study period or included in a sample. By contrast, case vignettes allow standardization, reproducibility, and targeted coverage of clinically important scenarios. However, the informativeness of vignette-based evaluations depends heavily on the case set used.

In this study, we build on both ideas by deriving vignettes from large-scale real-world triage encounters. More specifically, we aimed to construct a human-manageable vignette set from routine triage data that retains broad presentation diversity while preserving an explicit link to the underlying patient population.

### Prior Work

#### Vignette Studies

Previous studies have often relied on case vignettes authored by medical professionals and based on real cases or adapted from teaching materials and the medical literature (eg, [11-15]). Such expert-written vignettes can provide high-quality medical content, but their limited number usually constrains the range of patient presentations represented. As a result, they may not adequately reflect the diversity of real-world presentations encountered by triage systems or symptom checkers.

To increase realism and breadth, other studies have sampled real patient cases and converted them into vignettes (eg, [16-18]). These approaches are more likely to capture broader clinical variation, but explicit evaluations of how well the resulting vignette sets reflect population-level characteristics, such as demographic distributions or symptom frequencies, are rare [17]. In addition, random sampling may omit rare but medically critical conditions while including many common, similar cases. Other works have leveraged entire electronic health record datasets available for a defined patient group [19], but such approaches are feasible only for limited cohort sizes.

More recently, large language models (LLMs) have been used to generate medical vignettes (eg, [20-23]). Although some of these studies focused on predefined disease areas (eg, [21,23]), none aimed to capture or analyze population-wide characteristics of patient presentations. Moreover, to our knowledge, prior vignette studies have generally not quantified the representativeness of individual vignettes in terms of the number of real-world patients represented by each vignette in the underlying population. Such information would be highly valuable, as it could support extrapolation of evaluation results, such as misclassification rates, from the vignette set to the expected number of affected patients in the population. Thus, an important gap remains: there is currently no established framework for deriving a human-manageable vignette set from large-scale patient cohorts while preserving broad presentation diversity and linking individual vignettes to explicit quantitative weights.

#### Clustering of Clinical and Demographic Data

Clustering of patient-level clinical and demographic data is well established in medical informatics and is central to our framework for identifying representative triage cases for vignette generation. Prior studies have clustered patients using a wide range of structured variables, including age, sex, diagnosis codes, laboratory values, vital signs, and pain scores [24-31]. Other studies used free-text or semistructured clinical documentation, such as interview and examination notes [32], triage notes [33], scheduler notes [34], and broader clinical notes [35].

Methodologically, the literature spans a broad range of clustering paradigms, including hierarchical clustering [26,32,35], k-means [27,29,30,32,35,36], mixture models [35,37], density-based methods such as density-based spatial clustering of applications with noise (DBSCAN) or hierarchical density-based spatial clustering of applications with noise (HDBSCAN) [35,38,39], and mixed data methods such as k-prototypes [28,31]. In addition, some studies proposed task-specific similarity measures or clustering workflows tailored to particular forms of clinical data [40,41]. When free-text or semistructured documentation is involved, embedding-based representations have increasingly been used to capture semantic similarity [32-34,39].

Applications range from identifying phenotypes or patient subgroups [28-31] to treatment recommendations [24], disease detection [27], symptom grouping [35], or operational tasks such as appointment scheduling [34] and report summarization [42]. In triage and emergency care settings, clustering has been applied to patient prioritization [25], triage categorization [43], and identification of high-risk emergency department profiles [28].

Our use of clustering differs in its primary objective. Rather than using clustering for phenotype discovery, prediction, or operational optimization, we use it to condense many similar triage assessments into a single representative case that serves as the basis for a human-readable vignette. To our knowledge, this use of clustering has received little attention. Kelly et al [36] described a related approach based on patient-reported outcome data, but that work focused on a small, disease-specific dataset and on interpretability rather than on generating representative, population-linked vignettes from a large-scale dataset.

#### Scope and Objectives

In this study, we used nationwide triage data comprising all patient contacts with the on-call medical service (116117 phone line or website) in Germany. Patients contacting this service typically seek care for medical problems they perceive as urgent but not immediately life-threatening. The resulting triage recommendations can nevertheless span a broad range, from emergency referral to non-urgent telemedical consultation.

The aim of this study was to develop a data-driven framework for generating medical vignettes that would

- Capture broad characteristics of most 116117 contacts, including demographic characteristics and symptom patterns;
- Include an explicit weight reflecting the number of underlying patient assessments represented by each vignette;
- Ensure explicit consideration of rare emergency cases given the serious potential consequences of their misclassification; and
- Remain manageable in size to enable subsequent medical expert review and quality control.

For the first objective, simple random sampling of real-world assessments would be an obvious starting point. However, it would tend to reproduce many common and highly similar presentations, whereas less frequent but clinically important patterns might be sampled only rarely or not at all. It would neither condense redundant assessments into a smaller set of reviewable representatives nor provide an explicit link between a vignette and the number of similar underlying assessments.

To achieve these objectives, we semantically clustered questionnaires from a large sample of real triage assessments and condensed each eligible cluster into a single representative case. The contribution of this study lies in integrating and validating established clustering and optimization methods within a medical informatics framework that transforms large-scale routine triage data into a compact set of clinically reviewable, population-linked vignettes. We compared alternative clustering paradigms and assessment representations to identify a suitable configuration for this task. Clustering allowed common and similar presentations to be condensed into representative cases while retaining broad presentation diversity. Because each representative assessment originated from a defined cluster, the number of underlying assessments, and thus each representative’s weight relative to the sample population, was known. Rather than simply selecting the real assessment closest to each cluster centroid, our optimization aimed to generate a representative assessment that reflected the dominant within-cluster characteristics and maximized information content. Controlled LLM prompting then converted these representative assessments into structured textual vignettes.

To the best of our knowledge, no prior study has described a framework for deriving a human-manageable set of representative vignettes from large-scale real-world triage data that achieves near-complete sample coverage and links each vignette to an explicit sample-based weight. This combination may enable more population-aware benchmarking and quality assurance of digital triage tools.

## Methods

### Framework Overview

 Our proposed framework for generating a condensed, reviewable set of population-linked case vignettes from nationwide triage data is shown in Figure 1. First, triage questionnaires from a random sample of 50,000 real-world assessments collected over one year were transformed into semantic embeddings and grouped by agglomerative clustering to identify groups of similar patient presentations. For clusters containing a sufficient number of assessments, one representative assessment was generated to serve as the basis for a single vignette. Each representative assessment was intended to capture the central characteristics of its cluster while also resembling a realistic patient case that responds to many triage system questions. To balance these 2 aims—high representativeness and high information content—we implemented a 2-phase optimization procedure based on simulated annealing. Because each representative assessment originated from a defined cluster, the number of underlying assessments, and thus the cluster’s weight relative to the sample population, was known. This enabled comparison of the distributions of demographics, symptoms, and triage recommendations between the representative cases and the full sample. To assess whether the clustering-based weights are transferable to the full population, we additionally performed a similarity-based sensitivity analysis using the full 1-year assessment dataset. Finally, to make the results easily interpretable by medical professionals, every representative assessment was converted into a structured textual vignette using controlled prompting of an LLM.

**Figure 1.**
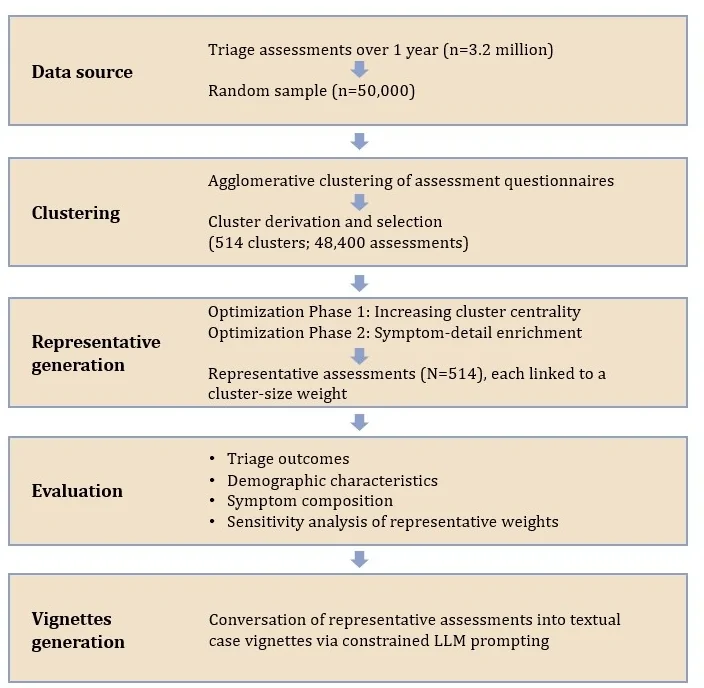
Overview of the study framework for generating population-linked case vignettes from nationwide triage data.

### Data Source

The data for this study originated from patient contacts with the German medical on-call service (116117) and from joint contact points of the outpatient emergency care service and hospital emergency departments (“Gemeinsamer Tresen” in German). Patients entered the triage service either by calling the hotline, where information was collected during the call; by completing an online self-assessment questionnaire; or by presenting in person at a joint contact point, where an on-site assessment was conducted. In all settings, versions of the same structured triage software based on clinical guidelines and published evidence were used. The system applies a standardized question flow, stores patient-reported information as an assessment, and generates a triage recommendation specifying the urgency of treatment and point of care. To account for seasonal effects, we considered all assessments recorded over a period of one year (November 1, 2023-October 31, 2024). From 3.2 million available assessments, we drew a simple random sample of 50,000 cases without replacement. No stratification by age, gender, or triage outcome was applied because the aim of this step was to obtain an unbiased but computationally manageable subset of the full 1-year dataset. Each assessment contained the following information: date of assessment, patient’s age, gender, pregnancy status if applicable; reported medical symptoms; responses to general health questions (eg, previous hospital visits, number of medications, preexisting conditions); and the triage outcomes time to treat (TTT) and point of care (POC). TTT comprised the categories “Emergency,” “As soon as possible,” “Within 24 hours,” “Not required within 24 hours,” and “Unclear or not available.” POC comprised “Emergency ambulance,” “Emergency room,” “Physician,” “Teleconsultation,” and “Unclear or not available.” Descriptive characteristics of the full 1-year dataset and the random sample are reported in the Results section. To compare the random sample with the full 1-year dataset, we reported effect size measures. For categorical variables, Cohen *w* [44] was used for the overall distribution and Cohen *h* [44] for pairwise differences in proportions. For count variables, we report Cohen *d* [44].

### Ethical Considerations

The data used in this study consist of pseudonymized individual-level social data that were lawfully collected and stored in accordance with Section 285(3) in conjunction with Section 285(1), number 6 of the German Social Code, Book V (SGB V), and may be processed for quality assurance purposes. The data are owned by the Associations of Statutory Health Insurance Physicians (ASHIPs). On the basis of contractual data processing agreements pursuant to Section 80 of the German Social Code, Book X (SGB X) in conjunction with Article 28 of the General Data Protection Regulation (GDPR), the Central Research Institute of Ambulatory Health Care in Germany (Zi) processes these data in pseudonymized form for quality assurance. Data transfer and processing were reviewed for compliance with applicable data protection regulations. Access to the data was restricted to authorized personnel.

### Clustering of Triage Assessments

To identify groups of assessments with similar patient-reported presentations, we performed a cluster analysis of a random sample of 50,000 assessments drawn from the 1-year population. This subsample was used because clustering algorithms become increasingly computationally demanding as the number of data points grows (eg, agglomerative clustering scales quadratically in both memory and runtime). The sample size was therefore chosen as a pragmatic compromise between population coverage and computational feasibility.

Clustering was based exclusively on question-answer information actively entered into the triage system; generated triage outcomes (TTT and POC) were excluded from the clustering features so that TTT could later be used as an external evaluation criterion.

We compared 3 clustering paradigms for grouping triage assessments: agglomerative clustering [45] (hierarchical clustering), k-means [46] (centroid-based clustering), and HDBSCAN [47] (density-based clustering) using 3 alternative assessment representations: one-hot encoding, one-hot encoding followed by principal component analysis (PCA) [48], and semantic embeddings derived from concatenated question-answer text using a sentence transformer model [49] specially trained on German text (“German Semantic STS V2” [50]). Approaches were evaluated with respect to 3 practical objectives: clinically meaningful grouping, broad assessment coverage, and a manageable number of clusters for subsequent vignette generation. As the primary application-oriented criterion, we used TTT homogeneity as an external proxy for clinical coherence; internal validity was additionally assessed using the silhouette coefficient [51], Calinski-Harabasz index [52], and Davies-Bouldin index [53]. Full details of the tested parameter combinations and comparative results are provided in Multimedia Appendix 1.

Agglomerative clustering provided the best trade-off between TTT homogeneity, assessment coverage, and the number of resulting clusters. The final configuration used semantic embeddings generated by “German Semantic STS V2” with Ward linkage and Euclidean distance. We selected the dendrogram cut corresponding to a TTT homogeneity of 0.5, as higher values led to substantially larger cluster sets and therefore many more vignettes. For representative assessment generation, we retained clusters containing at least 30 assessments. In addition, because emergency presentations were of particular relevance despite their lower frequency, we also included clusters composed exclusively of emergency assessments (ie, clusters in which all assessments had a POC of “Emergency ambulance,” or “Emergency room”).

### Generation of Representative Assessments

After identifying clusters of similar assessments, we aimed to construct one representative assessment per cluster. This representative case was intended to, first, reflect the central characteristics of the cluster and, second, to contain as much information as possible, that is, to include as many answered triage-system questions as possible without losing representativeness. Emphasizing the second criterion ensured that the resulting assessment not only captured dominant characteristics of the cluster but also resembled a plausible case for which many questions of the triage system could be answered. To achieve these objectives, a 2-phase simulated annealing procedure was implemented (details are provided in Multimedia Appendix 2).

### Evaluation of Representative Assessments

#### Agreement With Cluster-Level Triage Urgency

Each representative assessment originated from an actual case within its cluster and was adjusted using the frequency distributions of the respective question-answer combinations, ensuring that all assessment characteristics occurred in the cluster. Nevertheless, different answer combinations can correspond to varying symptom patterns and severity levels. Therefore, a reliable representative assessment should not only capture dominant symptom patterns of its cluster but also reflect the cluster’s majority triage recommendations, that is, the dominant treatment urgency. To evaluate this, each representative assessment was processed through the same triage system used for the original assessments by automatically submitting the corresponding question-answer pairs through the system’s application programming interface. The resulting triage outcomes (eg, TTT) of each representative assessment were recorded and compared with the TTT distribution of the originating cluster.

#### Symptom Coverage and Symptom Category Mapping

The triage system recorded information on 121 distinct symptoms. To evaluate how well the representative assessments covered the symptom spectrum observed in the original data, these symptoms were grouped into 13 broader symptom categories, loosely following the structure of the *International Classification of Diseases, 10th revision, German Modification* (*ICD-10 GM*) [54] and reviewed by a medical expert. Each symptom was assigned to one most suitable category. No suitable mapping could be identified for the general symptoms “Fatigue,” “Feeling unwell,” and “Crying baby/toddler.” The complete mapping is provided in Table S1 in Multimedia Appendix 3.

#### Sensitivity Analysis of Gender Effects

As an additional sensitivity analysis, we examined whether recorded gender influenced triage outcomes among representative assessments without gender-specific a priori content. Representative assessments were classified as gender-specific a priori if they contained symptoms or questionnaire content specific to one gender. The definitions used for this classification are provided in Multimedia Appendix 4. For all remaining non-gender-specific representatives, we created a gender-modified version by changing the recorded gender to male or female while preserving the remaining question-answer pairs. Each gender-modified assessment was then resubmitted to the triage system, and the resulting triage outcomes were compared with those of the original representative assessments.

#### Sensitivity Analysis of Vignette Weights

In our primary approach, each representative assessment and its resulting vignette were directly linked to a cluster of real-world assessments of known size, thereby providing a native cluster-based weight within the 50,000 assessment sample. However, this linkage may become less directly interpretable after medical expert review of the vignettes or in changing patient populations and is not transferable to external vignette sets. Therefore, we conducted a similarity-based sensitivity analysis that estimated vignette weights in the 1-year population of approximately 3.2 million assessments without relying on the original cluster-based weights. Representative assessments and population assessments were embedded using the “German Semantic STS V2” sentence transformer model, and vignette weights were re-estimated based on semantic similarity while avoiding double counting of assessments matched to multiple vignettes. Full methodological details are provided in Multimedia Appendix 2.

### Conversion of Representative Assessments Into Textual Case Vignettes

After identifying one representative assessment per cluster, we converted each into a structured German text vignette using GPT-4.1 (OpenAI) [55] under controlled prompting. The model was instructed to use only the provided question-answer content, include all answered questions, and organize the output into sections covering general patient information, medical history, and other medically relevant information. Interval-based expressions were converted into specific values using prompted sampling rules or through algorithmic preprocessing. The full prompt and preprocessing details are provided in Multimedia Appendix 5.

To assess adherence to the prompt instructions, we manually reviewed 50 randomly selected case vignettes against their underlying representative assessments (761 question-answer pairs in total). Twenty vignettes were reviewed by the author (AS) and 15 each by the authors (ES and JS) for hallucinated, misrepresented, and omitted information.

## Results

### Study Sample

From the full 1-year dataset of triage assessments recorded by the German medical on-call service, we drew a simple random sample of 50,000 assessments for clustering and representative assessment generation. As shown in Table 1, the sample closely resembled the full 1-year dataset in terms of demographic characteristics, triage recommendations, and triage questionnaires. Effect sizes were small in magnitude, suggesting limited distributional differences between the full 1-year dataset and the random sample (Cohen *w*=0.072 for gender, 0.077 for age, 0.023 for TTT, and 0.035 for POC).

**Table 1.** Demographic and triage-system characteristics of the full 1-year assessment dataset and the random sample of 50,000 assessments.

Characteristics	1-year (n=3,166,727)	Sample (n=50,000)	Effect size
Demographics			
Sex, n (%)			Cohen *w*=0.072
Male	1,310,551 (41.4)	21,120 (42.2)	Cohen *h*=0.017
Female	1,820,554 (57.5)	28,692 (57.4)	Cohen *h*=0.002
Not available	35,622 (1.1)	188 (0.4)	Cohen *h*=0.090
Age, n (%)			Cohen *w*=0.077
1‐4 weeks	4735 (0.1)	82 (0.2)	Cohen *h*=0.004
5‐8 weeks	5660 (0.2)	92 (0.2)	Cohen *h*=0.001
3‐12 months	55,613 (1.8)	873 (1.7)	Cohen *h*=0.001
1‐3 years	157,942 (5.0)	2414 (4.8)	Cohen *h*=0.007
4‐8 years	139,096 (4.4)	2190 (4.4)	Cohen *h*=0.001
9‐13 years	74,190 (2.3)	1114 (2.2)	Cohen *h*=0.008
14‐49 years	1,191,704 (37.6)	19,790 (39.6)	Cohen *h*=0.040
50‐65 years	471,641 (14.9)	7375 (14.8)	Cohen *h*=0.004
66‐80 years	477,452 (15.1)	7372 (14.7)	Cohen *h*=0.009
>80 years	548,984 (17.3)	8448 (16.9)	Cohen *h*=0.012
Not available	39,710 (1.3)	250 (0.5)	Cohen *h*=0.083
Triage outcomes			
TTT^a^, n (%)			Cohen *w*=0.023
Emergency	224,443 (7.1)	3745 (7.5)	Cohen *h*=0.015
As soon as possible	1,379,244 (43.6)	21,966 (43.9)	Cohen *h*=0.008
Within 24 hours	959,591 (30.3)	14,929 (29.9)	Cohen *h*=0.010
Not required within 24 hours	444,232 (14.0)	7010 (14.0)	Cohen *h*<0.001
Unclear or not available	159,217 (5.0)	2350 (4.7)	Cohen *h*=0.015
POC^b^, n (%)			Cohen *w*=0.035
Emergency ambulance	158,267 (5.0)	2696 (5.4)	Cohen *h*=0.018
Emergency room	809,130 (25.6)	13,324 (26.6)	Cohen *h*=0.025
Physician	1,502,027 (47.4)	23,373 (46.7)	Cohen *h*=0.014
Teleconsultation	531,630 (16.8)	8153 (16.3)	Cohen *h*=0.013
Unclear or not available	165,673 (5.2)	2454 (4.9)	Cohen *h*=0.015
Triage questionnaires			
Symptoms			
Available (n)	121	121	—^c^
Selected, mean (SD)	1.7 (1.3)	1.7 (1.3)	Cohen *d*=0.004
Questions			
Answered, mean (SD)	16.0 (9.5)	16.1 (9.5)	Cohen *d*=0.009

a TTT: time to treat.

b POC: point of care.

c Not applicable.

### Clustering Results

#### Overview

We evaluated several approaches to cluster the medical assessments in the sample, each represented by a set of question-answer pairs recorded in the triage system. Specifically, we compared agglomerative clustering, k-means, and HDBSCAN across different feature representations and, where applicable, distance metrics and hyperparameter settings. Clustering quality was evaluated using TTT homogeneity as a proxy because assessments with similar medical patterns should yield similar recommendations for treatment urgency. The best-performing approach was agglomerative clustering, which achieved a TTT homogeneity score of 0.5 with fewer clusters than k-means, whereas HDBSCAN either yielded lower TTT homogeneity or left a large proportion of assessments unclustered. The following section presents the outcomes of the best-performing configuration. Detailed results for all tested clustering configurations are reported in Multimedia Appendix 1, together with a sensitivity analysis assessing the robustness of the best-performing configuration across different sample sizes.

#### Dendrogram Characteristics

The best-performing clustering configuration was agglomerative clustering using semantic embeddings from the sentence transformer model “German Semantic STS V2,” combined with Ward linkage and Euclidean distance. The resulting dendrogram is shown in Figure 2. We analyzed the main branches (indicated by the dashed line) with respect to the distribution of reported symptoms and potentially urgent/emergency complaints recorded during the pretriage phase in the underlying assessments. The first 2 branches contain discontinued or incomplete assessments without triage recommendation or recorded symptoms (Branch 1, left unbranched child of the root: number of assessments [n_ass]=487, Branch 2: n_ass=1899). The remaining branches correspond to distinct thematic topics:

- Branch 3: musculoskeletal and cardiovascular complaints, led by lumbar back pain, leg problems, and dizziness (n_ass: 10,100);
- Branch 4: digestive-tract symptoms, led by vomiting/nausea, stomach pain, urinary tract complaints, and diarrhea (n_ass: 9637);
- Branch 5: eye complaints, led by eye redness, eye pain, and vision defect (n_ass: 3035);
- Branch 6: common cold-like symptoms, led by fever, cough, and influenza infection (n_ass: 11,610);
- Branch 7: comprised a right subbranch with skin problems and injuries, led by rash, wound/skin injury, accident/trauma, and itching, and a left subbranch with mental health issues, led by depressive feeling and anxiety (n_ass: 9434); and
- Branch 8: acute and potential emergency conditions, many identified during pretriage, such as cardiovascular, respiratory, and neurological complaints (n_ass: 3798).

**Figure 2.**
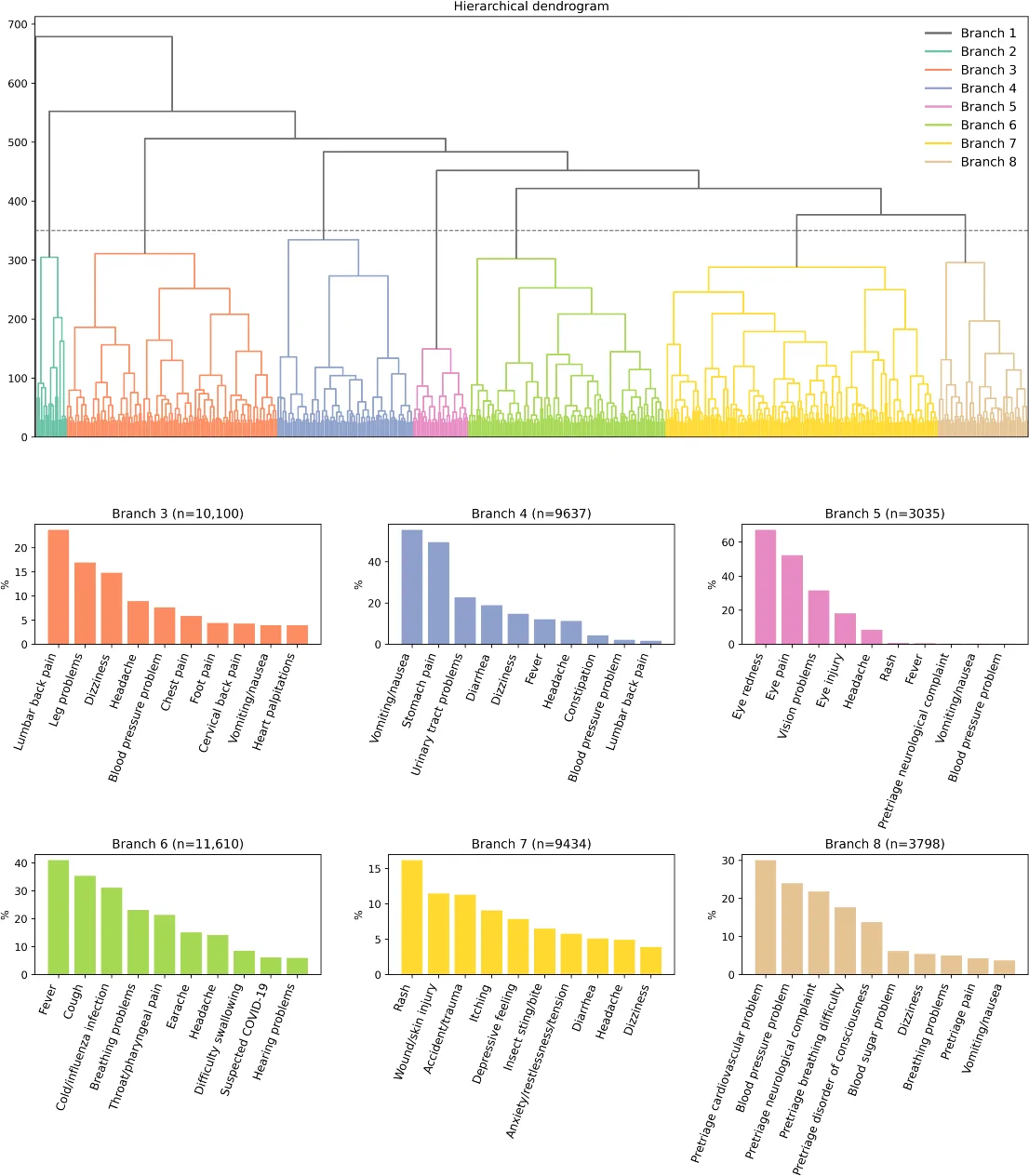
Main branches of the agglomerative clustering of triage assessments and their predominant complaint composition. The hierarchical clustering dendrogram of the 50,000 sampled assessments was cut at height 350 to define the color-coded main branches. For each main branch, the 10 most frequent recorded symptoms and pretriage complaints are shown.

These findings suggest that semantic embedding-based clustering produced medically interpretable groupings.

Figure 3 shows the proportion of each TTT category per dendrogram branch. It can be observed that many branches were dominated by a certain TTT category (eg, “Emergency” and “As soon as possible” assessments in main branch 8, which contained serious problems identified during pretriage), even though TTT was not part of the clustering features. This suggests that semantically similar assessments tended to have comparable treatment urgencies.

**Figure 3.**
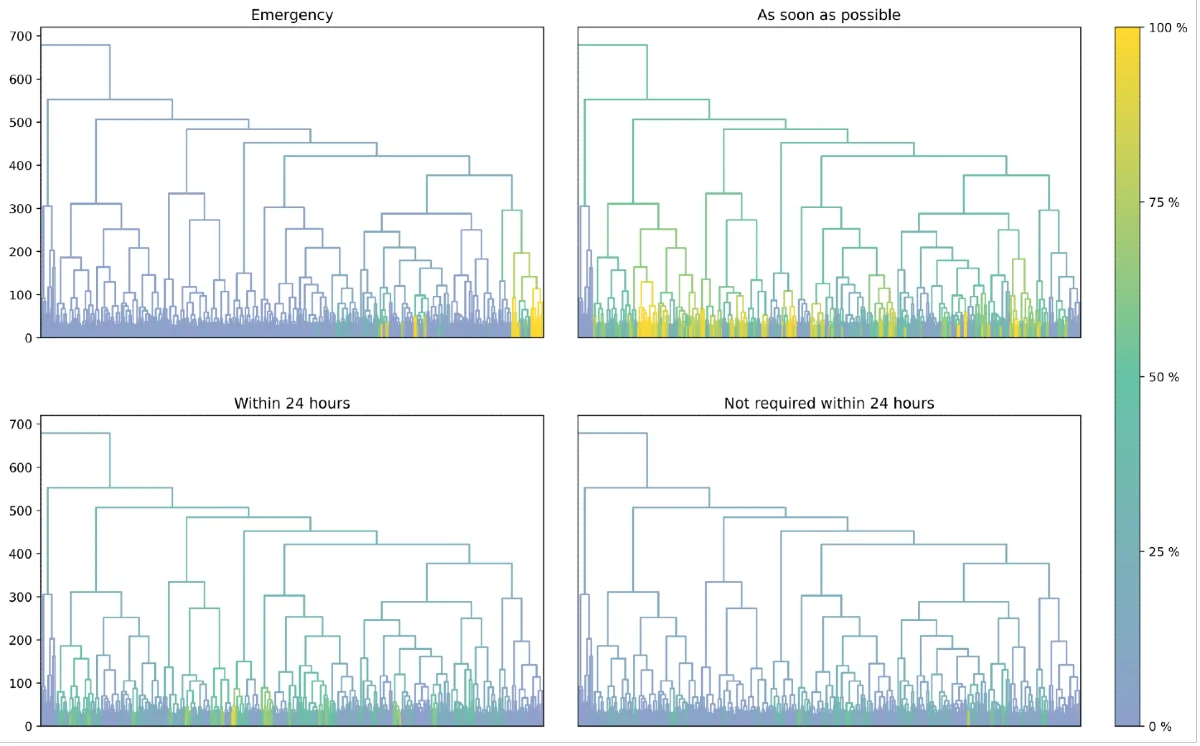
Distribution of time to treat (TTT) categories across dendrogram branches. For each TTT category with explicit urgency assignment, one dendrogram is shown, with branches color-coded according to the proportion of assessments in the respective branch assigned to that TTT category.

#### Cluster Characteristics

The dendrogram was cut at a threshold yielding a TTT homogeneity score of 0.5 (at height 23), resulting in 593 clusters. The assessments were well distributed across clusters; the number of assessments contained in each cluster (median 65, IQR 38‐102; minimum-maximum 6-487) is shown in Figure S4 in Multimedia Appendix 1. To focus on sufficiently large clusters for representative generation, we considered only clusters containing at least 30 assessments (498 clusters, 48,143 assessments). Increasing this threshold would have reduced sample coverage substantially (see Table S2 in Multimedia Appendix 1). Although emergency cases were less frequent, they were of particular medical relevance. Therefore, clusters composed entirely of emergency assessments were additionally included, adding 16 clusters comprising 257 assessments. In total, this resulted in 514 clusters, collectively containing 48,400 assessments that represented 96.8% of the original sample. For each of these 514 clusters, we have generated a representative assessment, the development steps of which are presented in the following section.

### Generation and Internal Evaluation of Representative Assessments

#### Overview

For each included cluster, one representative assessment was derived using a 2-phase optimization procedure. Phase 1 focused on generating an assessment that captured the central characteristics of the source cluster by minimizing the distance to the cluster centroid. Phase 2 then enriched this assessment with additional symptom-specific details while retaining the symptom set identified in Phase 1. The following analyses evaluate how these representative assessments developed across both optimization phases and how well they reflected their source clusters in terms of their triage urgency.

#### Development Phases

For each of the 514 included clusters, the real assessment closest to the cluster centroid was selected as the starting point for representative generation. In Phase 1, the optimization procedure generated a new artificial assessment that was closer to the centroid than the initial assessment in 346 (67.3%) clusters. In the remaining 168 (32.7%) clusters, the centroid-closest real assessment already represented the best solution. Among all 514 clusters, 458 (89.1%) yielded representative assessments that progressed beyond the pretriage stage and included at least one symptom profile, making them eligible for the enrichment step in Phase 2. In this second phase, a more comprehensive representative assessment was identified for 289 clusters (56.2% of all clusters), which remained close to the cluster centroid despite the added information, while 225 clusters (43.8% of all clusters) retained their Phase 1 representation. Overall, the final set of representative assessments comprised 126 (24.5%) unchanged real assessments corresponding to the initial centroid-closest case and 388 (75.5%) algorithmically generated assessments derived through optimization Phase 1 and/or Phase 2. These findings indicate that the centroid-closest real assessment often provided a useful starting point, but in most clusters, the optimization procedure generated a representative assessment that was either more centrally located within the cluster and therefore likely to better reflect cluster-level characteristics, more information-rich, or both.

#### Cluster Centrality and Information Content

To assess cluster centrality and information content, we compared the original cluster assessments, the real centroid-closest assessments, and the generated representative assessments obtained after Phase 1 and 2 (see Figure 4).

**Figure 4.**
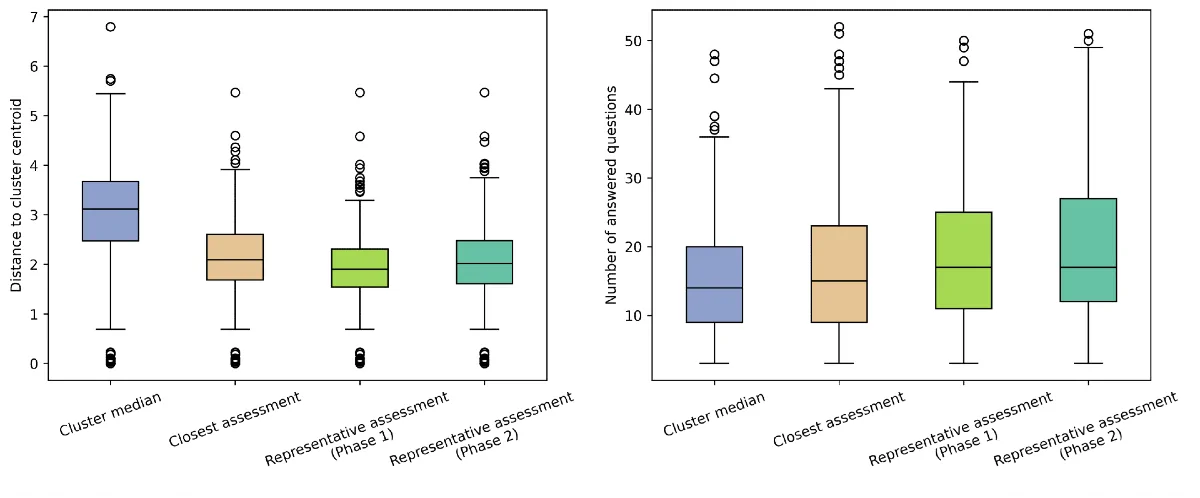
Evolution of representative assessments in terms of distance to the cluster centroid (left) and number of triage questions answered (right). For each cluster, the median value among the original assessments was compared with the centroid-closest real assessment and its development over optimization Phase 1 and Phase 2. Phase 1 reduced the distance to the cluster centroid, whereas Phase 2 increased the number of answered questions while largely preserving cluster centrality.

Across clusters, the mean of the median distances of the original assessments within each cluster to their respective centroids was 3.1 (SD 1.0). Selecting the centroid-closest real assessment reduced this distance to a mean of 2.1 (SD 0.7). After Phase 1, this mean distance further decreased to 1.9 (SD 0.6), indicating that the first optimization phase generally improved cluster centrality relative to the closest real assessment available in the cluster. Following Phase 2, the mean distance increased slightly to 2.1 (SD 0.7), showing that the enrichment step resulted in only a modest loss of centrality.

At the same time, information content—quantified as the number of answered triage questions—increased progressively over the 2 optimization phases. Across clusters, the median number of answered questions among the original assessments was a mean of 15.1 (SD 8.1). The initial centroid-closest real assessment contained a mean of 16.7 answered questions (SD 9.9). This number increased to 18.1 (SD 9.1) after Phase 1 and 19.4 (SD 10.0) after Phase 2. Compared with the median number of answered questions among the original assessments within each cluster, the final representative assessments therefore contained on average 4.3 additional answered triage questions.

Taken together, these results indicate that the 2-phase optimization procedure achieved the intended trade-off between cluster centrality and information content.

#### Agreement With Cluster-Level Treatment Urgency

To examine whether the representative assessments preserved clinically relevant urgency patterns of their source cluster, each representative assessment was processed through the triage system, and its TTT recommendation was compared with the distribution of TTT recommendations in its originating cluster.

Table 2 summarizes, for each TTT category, the number of clusters in which each TTT category represented the majority recommendation (the proportion of assessments assigned to the majority TTT categories varied across clusters), the TTT recommendations of the initial real assessments closest to the cluster centroids, and the number of representative assessments from Phase 1 and 2 receiving the same TTT recommendation. The overall distribution of TTT categories among the representative assessments closely resembled the distribution of cluster-level majority TTT categories. The largest absolute deviation was 2.9% for the category “As soon as possible.” When TTT categories were ordinally encoded, the TTT of the final representative assessments (Phase 2) showed a strong positive rank correlation with the cluster’s mean ordinal TTT (Spearman ρ=0.78; *P*<.001).

**Table 2.** Distribution of cluster-level majority TTT^a^ categories and TTT recommendations of the initial and representative assessments.

TTT	Cluster-level majority (N=514), n (%)	Initial centroid-closest assessment (N=514), n (%)	Representative assessment, Phase 1 (N=514), n (%)	Representative assessment, Phase 2 (N=514), n (%)
Emergency	49 (9.5)	54 (10.5)	45 (8.8)	49 (9.5)
As soon as possible	275 (53.5)	229 (44.6)	269 (52.3)	290 (56.4)
Within 24 hours	135 (26.3)	151 (29.4)	140 (27.2)	121 (23.5)
Not required within 24 hours	41 (8.0)	65 (12.6)	39 (7.6)	39 (7.6)
Unclear or not available	14 (2.7)	15 (2.9)	21 (4.1)	15 (2.9)

a TTT: time to treat.

Together, these findings suggest that the optimization procedure largely preserved the treatment-urgency patterns of the originating clusters.

### Population-Level Representativeness

#### Overview

In this section, we evaluated the extent to which the final representative assessments reproduce key characteristics of the 50,000 sampled medical on-call assessments. The 514 clusters used for generating representatives collectively contained 48,400 assessments, corresponding to 96.8% of the sample. To assess potential selection bias due to non-represented clusters, we compare the overall sample distributions with those of the 48,400-assessment subset. Because each representative assessment was derived from a single cluster, the representatives were weighted by the number of assessments contained in their source cluster. For each investigated characteristic, we additionally report the proportion of assessments belonging to clusters in which that characteristic formed the majority value, as an indicator of how strongly it was clustered.

#### Triage Outcomes

We applied the same triage system from which the original assessments were obtained to the generated representative assessments and compared the resulting triage recommendations with those of the full sample and among the assessments in the selected clusters. Overall, the distributions of TTT and POC were highly similar between the full sample and the subset of assessments contained in the selected clusters, indicating that exclusion of smaller nonselected clusters introduced only little distortion with respect to these outcomes.

In the cluster-size-weighted representative assessments, however, a moderate shift toward higher-urgency categories was observed (see Table 3). Compared with the full sample, the proportion classified as “As soon as possible” increased by 6.6 percentage points (50.5% vs 43.9%), and “Emergency room” recommendations increased by 7.2 percentage points (33.8% vs 26.6%). In contrast, the lower-urgency recommendations “Not required within 24 hours” and “Teleconsultation” were underrepresented by 6.3 percentage points (7.7% vs 14.0%) and 6.5 percentage points (9.8% vs 16.3%), respectively.

**Table 3.** Comparison of triage outcomes of the full 50,000 assessment sample, the assessments within included clusters for representative generation (48,400 assessments), and the weighted set of the final representative assessments.

Triage outcome	Sample (n=50,000), n (%)	Assessments in selected clusters (n=48,400), n (%)	Weighted representative assessments (n=48,400), n (%)	Assessments in clusters in which the category was the majority, n/N (%)
TTT^a^				
Emergency	3745 (7.5)	3594 (7.4)	2942 (6.1)	2548/3594 (70.9)
As soon as possible	21,966 (43.9)	21,139 (43.7)	24,432 (50.5)	16,811/21,139 (79.5)
Within 24 hours	14,929 (29.9)	14,745 (30.5)	14,828 (30.6)	10,404/14,745 (70.6)
Not required within 24 hours	7010 (14.0)	6792 (14.0)	3731 (7.7)	2468/6792 (36.3)
Unclear or not available	2350 (4.7)	2130 (4.4)	2467 (5.1)	1978/2130 (92.9)
POC^b^				
Emergency ambulance	2696 (5.4)	2550 (5.3)	2057 (4.3)	1993/2550 (78.2)
Emergency room	13,324 (26.6)	13,027 (26.9)	16,346 (33.8)	8086/13,027 (62.1)
Physician	23,373 (46.7)	22,650 (46.8)	22,620 (46.7)	18,683/22,650 (82.5)
Teleconsultation	8153 (16.3)	7939 (16.4)	4745 (9.8)	3305/7939 (41.6)
Unclear or not available	2454 (4.9)	2234 (4.6)	2632 (5.4)	1978/2234 (88.5)

a TTT: time to treat.

b POC: point of care.

To help interpret these deviations, we additionally calculated, for each TTT and POC category, the proportion of assessments belonging to clusters in which that category constituted the majority value. High proportions were observed for “Physician” (82.5%), “As soon as possible” (79.5%), and “Emergency ambulance” (78.2%), indicating that these categories formed relatively coherent cluster majorities. In contrast, only 36.3% of assessments with the recommendation “Not required within 24 hours” belonged to clusters dominated by this category, which may partly explain its underrepresentation in the weighted representative assessments.

#### Demographic Characteristics

The gender distribution of the 48,400 assessments underlying the representative assessments was nearly identical to that of the full 50,000-assessment sample (see Table 4). In contrast, the cluster-size-weighted representative assessments represented 13.5% more female cases than the original sample. Female assessments were more frequent than male assessments in the sample (57.4% vs 42.2%), and 84% of female assessments belonged to clusters in which females formed the majority, compared with 38% for male assessments. As a result, clusters with theoretically gender-neutral symptom patterns tended to contain more female cases, making them more likely to serve as representatives and, when aggregated across clusters, leading to an overall shift toward female assessments in the weighted representative set.

**Table 4.** Comparison of demographic characteristics of the full 50,000 assessment sample, the assessments within included clusters for representative generation (48,400 assessments), and the weighted set of the final representative assessments.

Demographics	Sample (n=50,000), n (%)	Assessments in selected clusters (n=48,400), n (%)	Weighted representative assessments (n=48,400), n (%)	Assessments in clusters in which the category was the majority, n/N (%)
Sex				
Male	21,120 (42.2)	20,416 (42.2)	13,607 (28.1)	7660/20,416 (37.6)
Female	28,692 (57.4)	27,800 (57.4)	34,306 (70.9)	23,432/27,800 (84.3)
Not available	188 (0.4)	184 (0.4)	487 (1.0)	182/184 (98.9)
Age				
1‐4 weeks	82 (0.2)	64 (0.1)	132 (0.3)	0/64 (0.0)
5‐8 weeks	92 (0.2)	65 (0.1)	0 (0.0)	0/65 (0.0)
3‐12 months	873 (1.7)	829 (1.7)	907 (1.9)	357/829 (43.1)
1‐3 years	2414 (4.8)	2343 (4.8)	2590 (5.4)	1266/2343 (54.0)
4‐8 years	2190 (4.4)	2089 (4.3)	1466 (3.0)	509/2089 (24.4)
9‐13 years	1114 (2.2)	1042 (2.2)	110 (0.2)	33/1042 (3.2)
14‐49 years	19,790 (39.6)	19,307 (39.9)	23,057 (47.6)	18,101/19,307 (93.8)
50‐65 years	7375 (14.8)	7122 (14.7)	5532 (11.4)	2045/7122 (28.7)
66‐80 years	7372 (14.7)	7142 (14.8)	5325 (11.0)	1509/7142 (21.1)
>80 years	8448 (16.9)	8164 (16.9)	8794 (18.2)	4899/8164 (60.0)
Not available	250 (0.5)	233 (0.5)	487 (1.0)	230/233 (98.7)

To further examine the implications of this gender imbalance for triage outcomes, we performed an additional gender-effects sensitivity analysis. Among representative assessments without a priori gender-specific content, changing the recorded gender altered triage outcomes in only 3 representatives, all of which concerned urinary-tract complaints and resulted in higher urgency in the male version. Details are reported in Supplementary Results in Multimedia Appendix 4.

The age distribution of the weighted representative assessments was overall similar to that of the sample, although some deviations were observed. The largest deviation occurred in the 14‐49-year group, which was overrepresented in the weighted representatives (8.0%). This likely reflects a similar mechanism as mentioned for the female cases, since this group forms the largest age segment in the sample (39.6%), and 93.8% of assessments in this age group belonged to clusters in which they formed the majority.

#### Symptom Coverage

During the sampling period, the triage system recorded information on 121 distinct symptoms. Of these, 101 (83.5%) were represented in at least one final generated representative assessment. The remaining, noncovered symptoms occurred in fewer than 0.5% of all sampled assessments. To assess broader symptom coverage, the symptoms were mapped to symptom categories loosely aligned with *ICD-10-GM* chapters (see section “Symptom Coverage and Symptom Category Mapping”). For each symptom category, the proportion of assessments that recorded at least one symptom from that category was determined for the total sample (n=50,000) and for the subset of representative clusters (n=48,400) (see “Sample” and “Subsample” in Figure 5). These frequencies were compared with the weighted proportions of representative assessments containing a symptom of the corresponding category (see “Weighted representative assessments” in Figure 5). Across most symptom categories, the weighted representative assessments closely approximated the sample frequencies. The largest deviation was observed for the category “Nervous system” (deviation 5.1%, sample 15.2%, weighted representatives 20.3%), mainly driven by the symptom “headache” (5.3% of the weighted representatives). All remaining symptom categories differed by less than 1%, indicating that the representative assessments closely reproduce the overall symptom distribution of the source data.

**Figure 5.**
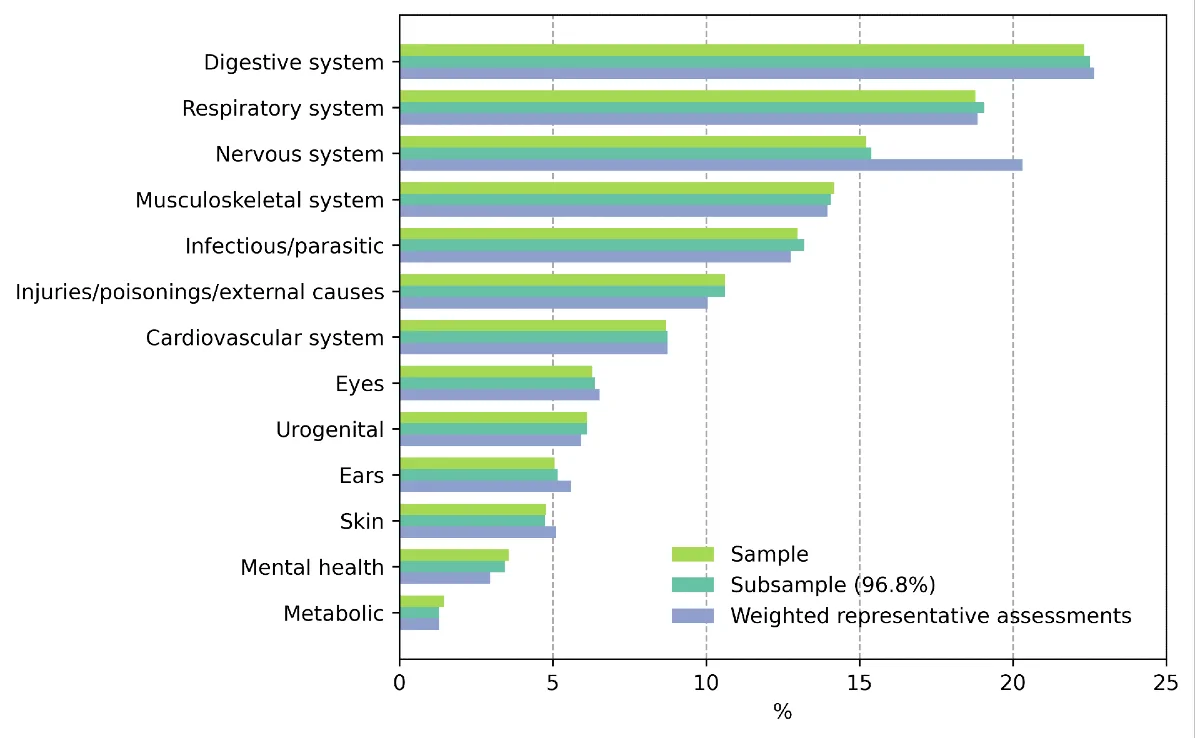
Comparison of the symptom category distributions of the full sample of 50,000 assessments, the assessments within included clusters for representative generation (subsample: 48,400 assessments), and the weighted set of the final representative assessments.

#### Sensitivity Analysis of Vignette Weights and Population Coverage

The analyses above evaluated how well the representative assessments reproduced key characteristics of the 50,000-assessment sample when weighted by cluster size. To examine whether these cluster-based, sample-derived vignette weights were preserved at the population level, we additionally applied a similarity-based sensitivity analysis to the full 1-year population of approximately 3.2 million assessments.

The resulting similarity-based vignette weights showed a strong positive rank correlation with the original cluster-based weights (Spearman ρ=0.77; *P*<.001), suggesting that the relative importance of individual vignettes was largely preserved from sample to population.

In the similarity-based analysis, 90.1% (2,853,281/3,166,727) of assessments in the 1-year population were matched to at least one vignette from the generated vignette set. As a reference, the original cluster-based approach linked the 514 representative assessments to 48,400 assessments, corresponding to 96.8% of the 50,000-assessment sample.

### Textual Case Vignettes

The representative assessments consisted of sets of question-answer pairs derived from a triage system. These data were transformed into structured medical case vignettes using controlled prompting of an LLM, making them more accessible for health care professionals.

The generated vignettes followed the instructed structure, which comprised patient information, medical history, and other medically relevant information. Textbox 1 presents an example vignette (translated from German); additional examples are provided in Multimedia Appendix 5. Information originally recorded by the triage system in interval formats (eg, age ranges, pain intensity, and symptom durations) was successfully converted into specific values (marked by underlining in the text) to simulate realistic patient presentations.

Textbox 1. **Example of a generated case vignette.**

The following points illustrate the structure and content of a representative case vignette generated from the corresponding representative assessment:

1. Patient: a 2-year-old boy.
2. Medical history (anamnesis): symptom complex (ear discharge, ear pain, and fever): For the past 2 days, fluid had been coming from the right ear, with no pus or blood visible. In addition, moderate right-sided ear pain (5 out of 10) had been present since that day. The parents report that the boy has a fever; the measured body temperature was 38.7  °C, and the fever had begun that day. Mild chills are reported as an accompanying symptom. The child is more restless than usual. Home remedies or over-the-counter medications have already been used for self-treatment. Chin function is normal; the chin can be moved toward the chest. There is no extreme dryness of the mouth, no marked thirst, and no restriction of urination. Inflammation of the auricle or the area around the ear is unclear. No blisters are visible on the auricle or around the ear.
3. Other medically relevant information: there was no immediately life-threatening situation. No relevant surgery has been performed within the past 14 days. No immune deficiency is known. The young patient had not been abroad recently. He was not currently taking any new medications, and within the past few weeks no new medication has been started, and no dosage of existing medications has been increased.

In a descriptive manual review of 50 randomly selected case vignettes against their underlying representative assessments (761 question-answer pairs in total), we identified 1 hallucinated statement and 4 misrepresentations (for details see Table S1 in Multimedia Appendix 5). We further observed 24 omissions, 21 of which concerned the preliminary triage item “Is there an acute life-threatening situation?” with the recorded response “No” or “Unclear.” These items were likely often omitted because the prompt instructed the LLM not to make assumptions, assessments, or diagnoses; when included, they will be removed during subsequent medical expert review because they are considered to constitute an unintended assessment of the patient’s condition.

## Discussion

### Principal Findings

This study presents an automated, data-driven framework for condensing a large volume of real triage data from the German medical on-call service (116117) into a human-manageable set of weighted case vignettes linked to clusters covering the vast majority of sampled patient contacts. From a random sample of 50,000 triage assessments, the best clustering performance was achieved using agglomerative clustering with semantic embeddings, resulting in 514 representative clusters that together covered 96.8% of the sample. The resulting dendrogram revealed medically interpretable groupings, indicating that semantic embedding-based clustering captured meaningful structure in this heterogeneous triage dataset.

To derive one representative case per cluster, we implemented a 2-phase optimization procedure that balanced proximity to the cluster centroid with information completeness. The resulting representative assessments were, on average, closer to their respective cluster centroids and contained more answered triage questions than the original assessments, indicating improved representativeness and detail. In addition, the TTT recommendations of the representative assessments were strongly correlated with the mean TTT of their source clusters, suggesting that clinically relevant triage patterns were largely preserved.

A key contribution of the framework is that each representative assessment remains linked to its source cluster of known size. This makes it possible to assign each vignette an explicit weight within the sample population. When weighted and aggregated across clusters, the representative assessments covered all nonrare symptoms captured by the triage system and the full spectrum of treatment urgency levels. Most demographic, symptom, and treatment urgency distributions were approximated reasonably well, although some systematic deviations remained. Most notably an overrepresentation of female cases, individuals aged 14‐49 years, and the urgency category “As soon as possible,” as well as an underrepresentation of the urgency category “Not required within 24 hours.” These deviations are important because they show that condensing each cluster into a single representative case preserves broad presentation diversity but does not fully retain all marginal distributions of the source population. Deviations in treatment urgency affect how closely the final vignette set reflects the original urgency mix, whereas demographic deviations may additionally influence the validity of subgroup-sensitive benchmarking if tool performance differs across demographic groups despite similar symptom presentations.

These deviations can be explained with the logic of building a single “best” representative case per cluster. Features that cluster well and therefore dominate certain clusters are more likely to be included in a representative assessment than features that are widely dispersed across clusters. For instance, only a fraction of assessments with the TTT category “Not required within 24 hours” formed cluster majorities, resulting in fewer representatives of this type than expected from the sample distribution. Similarly, characteristics that are frequent in the source population may become overrepresented because they more often form cluster majorities and are therefore more likely to be selected in a representative case. Gender provides an example: females were more common in the sample and therefore more often dominated clusters whose defining symptom patterns were not intrinsically gender-specific, thereby increasing the probability that female cases would be selected as representatives. A similar mechanism likely contributed to the overrepresentation of individuals aged 14‐49 years, which constituted the largest age segment in the source sample and most often formed cluster majorities, whereas smaller pediatric and older adult groups were less often selected as representatives of symptom-defined clusters.

To better understand the practical implications of the demographic distortion, we performed an additional sensitivity analysis for representative assessments without a priori gender-specific content. For the evaluated triage system, this suggests that the observed gender imbalance primarily affected the demographic realism of the vignette set rather than urgency assignment for most non-gender-specific presentations. However, this should not be generalized to other triage systems or diagnostic tasks.

To assess whether the cluster-based weights were transferable to the full 1-year population, we performed an additional similarity-based sensitivity analysis. The findings suggested that both broad coverage and the relative importance of individual vignettes were largely preserved at the population level.

Finally, the question-answer pairs of the representative assessments were transformed into structured textual case vignettes using a controlled LLM-prompting approach. In this workflow, the LLM was not used to generate case content de novo but to verbalize information already present in the representative assessments. This step translated triage data into concise, readable case descriptions that may facilitate medical expert review and further development.

### Comparison With Prior Work

Placed in the broader context of research on clinical vignettes, this study differs from previous work in several aspects. Many prior vignette studies relied on physician-authored vignettes (eg, [12,14,15]), which can ensure high medical quality but limit the range of clinical presentations represented. Others deliberately focused on predefined disease areas (eg, [21,56]), thereby narrowing their coverage by design. Approaches that came closer to covering a broad spectrum of medical conditions typically deployed random sampling of real patient cases (eg, [16-18]). However, only a few explicitly considered population distributions of symptoms or demographics when selecting vignette samples. For example, Kopka et al [17] accounted for the distribution of symptom categories when selecting cases from Reddit (Reddit, Inc) posts, representing an exception among largely unweighted sampling strategies.

In contrast, our study used an automated data-driven framework to condense real large-scale triage data into a human-manageable number of representative cases and explicitly evaluated how well key population characteristics were retained. In addition, each generated case remained linked to a quantitative population weight, an aspect that, to our knowledge, has not been addressed in prior vignette studies. This is relevant because explicit weighting may allow vignette-based evaluation results, such as undertriage or overtriage, to be extrapolated to the expected number of affected patients in the underlying population.

With the increasing capabilities of LLMs in recent years, recent studies introduced LLMs into their vignette generation workflow. Some used LLMs primarily to structure or formulate existing medical information (eg, [57,58]), whereas others used them to generate vignette content for predefined diseases (eg, [20,21,23]). Our study belongs to the first category: a representative triage assessment was supplied as factual input, and the LLM was used solely to structure and verbalize this information into medical vignettes. In this sense, our approach illustrates how real data-grounded content and the linguistic capabilities of LLMs can complement each other.

### Limitations

The following limitations should be considered when interpreting the findings of this study.

The generated vignettes are based on assessments from the German medical on-call service (116117) and therefore reflect only patients who accessed this service rather than all patients with acute health concerns in Germany. Use of 116117 may depend on factors such as awareness of the service [59], help-seeking preferences, and access to or ability to use telephone, web-based, or on-site triage services, which may limit representativeness for some patient groups. In addition, the vignettes are constrained by the information captured by the triage system. This includes predefined symptom lists, their permitted combinations, the level of detail recorded for each symptom, and more general patient information such as preexisting conditions or medication use. Many responses are also recorded only in broad categories (eg, age ranges, vital-sign intervals, and duration of symptoms).

Another limitation concerns potential reporting bias in the underlying triage data. We did not formally assess whether some users tested the system without an actual acute health concern, misunderstood questions, or strategically overstated symptom severity. Such risks may differ between triage settings; telephone triage involves trained dispatchers who can ask follow-up questions and perform some real-time plausibility checks, whereas online self-triage is more susceptible to exploratory or strategic use. Accordingly, the vignettes should be interpreted as reflecting recorded triage presentations rather than clinically validated ground truth. Related ongoing work in the German 116117 context, such as “Identification of emergencies in the telephone queue and routing to a fast track (FAST)” [60], addresses broader questions of urgency self-rating and possible misuse incentives in queue management but does not directly validate the present dataset.

The triage system itself is designed to provide rapid, rule-based urgency recommendations rather than capturing complete medical patient histories. As a result, emergency assessments in particular are intentionally short and focused on the core problem to avoid delays in patient treatment. Consequently, the resulting vignettes may lack the narrative richness of real patient encounters.

Condensing each cluster into a single representative case reduces intracluster variance and may underrepresent rare or atypical presentations. It can also distort marginal demographic distributions. In our study, this was most visible in the overrepresentation of female cases and individuals aged 14‐49 years. Such distortions may matter when benchmarking tools whose recommendations vary systematically by demographic group. However, simple demographic reweighting is not straightforward because vignette weights are linked to sets of triage question-answer patterns and are intended to reflect the frequency of underlying patient presentations in the source population rather than independently sampled patient cases. Reweighting by demographic variables alone could therefore improve demographic alignment while weakening the correspondence between vignette weights and the underlying distribution of presentation patterns, symptom constellations, and urgency patterns. Moreover, whereas a counterfactual sensitivity analysis was feasible for non-gender-specific representatives, an analogous analysis was not performed for age because age directly constrains symptom eligibility and questionnaire pathways in the triage system, making isolated age changes difficult to simulate in a medically plausible way.

In addition, generating a representative assessment close to the cluster centroid may combine characteristics of the cluster that seldom co-occur in real patients. More broadly, semantic similarity in the embedding space does not necessarily imply full medical equivalence.

These limitations were also reflected in a preliminary medical expert review of the generated vignettes. Some cases lacked contextual details that would help clinicians assess them with greater confidence, such as precise durations of symptoms, the circumstances of an accident, or the nature of a wound, as well as overly generalized information originating from the triage system that could represent distinct clinical conditions if specified individually, and some vignettes showed logical inconsistencies, such as contradictory timelines between related symptoms or events or conflicting information on risk factors. These observations highlight that data-driven representativeness does not automatically guarantee full clinical coherence at the individual vignette level.

The LLM was prompted under strict constraints to avoid hallucinations and to include all available information from the selected question-answer pairs. However, complete control over the generation process cannot be guaranteed.

Finally, because the data used in this study originate from a single national triage system, the extent to which the vignettes generalize to other health care settings remains to be evaluated.

### Implications and Future Directions

We condensed thousands of triage assessments into a human-manageable set of 514 representative cases that together characterize almost the entire sample population. Each case vignette is linked to a specific number of underlying assessments, providing a sample-based weight that supports weighted evaluation frameworks. This kind of linkage between vignettes and underlying assessment frequency has, to our knowledge, not been available in previous vignette datasets.

The resulting large and diverse dataset of vignettes may provide a useful basis for studying differences in triage or diagnostic behavior across various digital decision-support tools, symptom checkers, or LLMs, all of which are increasingly used in medical contexts [1-3] and may therefore affect a growing number of patients. The large number of vignettes may enable more stratified comparison than smaller vignette sets. Future releases of the vignette set could improve transparency by providing, for each vignette, the gender and age composition of the underlying source cluster. This information could be used to complement overall weighted analyses with subgroup-aware reporting, without distorting the native cluster-based weights or altering the underlying cluster definitions. Applying the vignettes to other digital triage systems may highlight limitations in our present narratives encapsulated in the question-answer sequences as generated by the on-call medical service in Germany. Enriching the present data basis may increase the value of the resulting set of vignettes.

Planned next steps include iterative physician review of all vignettes to improve their clinical coherence and validity, followed by physician triage of the finalized set by 5 medical experts. Each vignette will be assessed for TTT and POC. In cases of disagreement or identified inconsistencies or missing details, the vignette will be revised and re-evaluated with the aim of achieving physician consensus. We aim to release the curated vignette set as a public benchmark dataset and enable the submission of triage tool results for evaluation against this medical expert consensus. These steps will not only enable systematic comparison between tools and human judgment but also allow assessment of potential risks such as under- or overtriage. Beyond benchmarking, the vignettes may also support physician education and further development of safe triage systems.

### Conclusions

This study presents a data-driven framework for generating a human-manageable set of population-linked case vignettes from nationwide triage data. Applied to a random sample of 50,000 triage assessments, the framework produced 514 weighted representative cases that covered most of the sampled presentations and approximated key distributions of demographics, symptom categories, and treatment urgency. The generated vignettes contribute to the diversity of existing vignette resources and may support more population-aware benchmarking of digital decision-support tools. Before publishing the vignette set, expert refinement is planned to strengthen medical coherence and quality.

## Supplementary material

10.2196/104801 Multimedia Appendix 1 Detailed clustering methods and evaluation of different clustering approaches.

10.2196/104801 Multimedia Appendix 2 Additional methods and results for representative assessment generation and vignette-weight sensitivity analysis.

10.2196/104801 Multimedia Appendix 3 Mapping of triage symptoms to symptom categories.

10.2196/104801 Multimedia Appendix 4 Supplementary methods and results of the gender-effects sensitivity analysis.

10.2196/104801 Multimedia Appendix 5 Supplementary methods and results regarding case vignette generation.

## Abbreviations

ASHIP: Association of Statutory Health Insurance Physicians
DBSCAN: density-based spatial clustering of applications with noise
GDPR: General Data Protection Regulation
HDBSCAN: hierarchical density-based spatial clustering of applications with noise
*ICD-10 GM*: *International Classification of Diseases, 10th revision, German Modification*
LLM: large language model
n_ass: number of assessments
PCA: principal component analysis
POC: point of care
SGB V: German Social Code, Book V
SGB X: German Social Code, Book X
TTT: time to treat
