# Supplementary material for: Generating Population-Linked Case Vignettes From Nationwide Triage Data: Framework Development and Validation Study
Source: JMIR Med Inform. 2026 Nov 3;14:e104801. doi: 10.2196/104801 (PMC13730294; doi:10.2196/104801)
Supplement: Supplementary file 1 [file medinform-v14-e104801-s001.pdf]

Multimedia Appendix

Detailed clustering methods and evaluation of different clustering approaches.

Supplementary Methods: Detailed Clustering Methods

Clustering Input

To identify groups of assessments with similar patient-reported presentations, we performed a cluster analysis on a random sample of 50,000 assessments drawn from the 1-year population. Clustering was based exclusively on the information actively entered into the triage system, namely the questions and their corresponding answers, while triage system generated outcomes (TTT and POC) were not used as clustering features. This separation was intended to ensure that the clustering reflects the semantic similarities in patient-reported information and allowed TTT to be used subsequently as an external criterion for evaluating cluster quality.

Selection Criteria for Clustering Approaches

In this study, a useful clustering approach needed to satisfy three practical objectives: it should group assessments in a clinically meaningful way, retain broad coverage of the sampled population, and yield a manageable number of clusters for subsequent vignette generation and expert review. We therefore evaluated clustering quality primarily using TTT homogeneity as an external proxy for clinically relevant grouping, as medically similar presentations would be expected to receive similar treatment-urgency recommendations. Additionally, since broad population coverage was a further objective, clustering approaches that left large proportions of assessments unclustered were considered less suitable. To complement these application-oriented criteria, we additionally assessed internal cluster validity using the silhouette coefficient [1], Calinski-Harabasz index [2], and Davies-Bouldin index [3].

Comparison of Clustering Paradigms

We compared three clustering paradigms for grouping triage assessments: agglomerative clustering [4] (hierarchical clustering), k-means [5] (centroid-based clustering), and Hierarchical Density-Based Spatial Clustering of Applications with Noise [6] (HDBSCAN; density-based clustering).

For each method, question-answer pairs were represented using one-hot encoding (3709 dimensions), one-hot encoding followed by principal component analysis (PCA) [7] retaining at least 90% of the variance (473 dimensions), or semantic embeddings generated from concatenated question-answer texts, with symptom-specific entries prefixed by the symptom name using the German Sentence Transformer model “German Semantic STS V2” [8] (1024 dimensions).

For agglomerative clustering, we performed a systematic grid search across all eligible combinations of feature representation, linkage method ("average", "complete", "ward"), and distance metric ("euclidean", "cosine", "jaccard"). Different distance metrics were evaluated because the most suitable notion of similarity was not known a priori and may depend on the underlying representation of the assessments. Jaccard distance was evaluated only for one-hot encoded representations, because it is intended for binary set-like data and is not meaningful for dense continuous vectors such as PCA scores or sentence embeddings. Ward linkage was evaluated only with Euclidean distance, because its variance-minimizing criterion is defined in Euclidean space. For each eligible configuration, clustering quality was assessed across a range of dendrogram cuts corresponding to 100 to 5000 clusters (see Figure S1 in this appendix). Clustering was performed using the cluster.hierarchy.linkage function from the SciPy package version 1.15.2 [9].

For k-means, which operates in Euclidean space and does not produce a hierarchical clustering structure, we evaluated the same three feature representations and fitted the algorithm independently for each candidate number of clusters from 100 to 5000 (see Figure S2 in this appendix) using the cluster.KMeans function from scikit-learn version 1.9.0 [10].

For HDBSCAN, we evaluated eligible combinations of representation and distance metric ("euclidean", "cosine", "jaccard"), again with Jaccard distance restricted to one-hot encoded data using the cluster.HDBSCAN function from scikit-learn version 1.9.0 (see Table S1 in this appendix).

Final Clustering Configuration

Among the evaluated approaches, agglomerative clustering was selected for subsequent representative assessment generation because it best balanced achieved TTT homogeneity with the number of clusters and assessments coverage. In contrast, k-means required more clusters to achieve the same TTT homogeneity, whereas HDBSCAN either produced lower TTT homogeneity or left a large proportion of assessments unclustered (see section "Supplementary Results: Evaluation of Different Clustering Approaches" in this appendix).

Within agglomerative clustering, we selected the final configuration as the one that achieved a TTT homogeneity score of 0.5 with the smallest number of clusters. Although higher homogeneity would be desirable in principle, this would result in substantially more clusters (with considerably fewer assessments per cluster) and consequently a much higher number of vignettes to be generated (e.g., a homogeneity of 0.6 would result in >2500 clusters, see Figure S1 in this appendix). A homogeneity threshold of 0.5 was therefore chosen as a trade-off between medical similarity of assessments within clusters and the number and size of clusters obtained. Under this selection criterion, the best clustering performance was

achieved using semantic embeddings from the Sentence Transformer model "German Semantic STS V2" [8] combined with Ward linkage and Euclidean distance.

For representative assessment generation, we retained clusters containing at least 30 assessments. This minimum cluster size was chosen as a pragmatic compromise between retaining broad sample coverage and ensuring that each retained cluster contained a meaningful number of assessments to build a representative. Higher minimum cluster-size thresholds would have reduced sample coverage substantially (see Table S2 in this appendix). Because emergency cases are of particular medical relevance but occurred less frequently in our sample, we additionally included clusters exclusively comprising emergency assessments (POC of all assessments in ["Emergency ambulance", "Emergency room"]).

Supplementary Results: Evaluation of Different Clustering Approaches

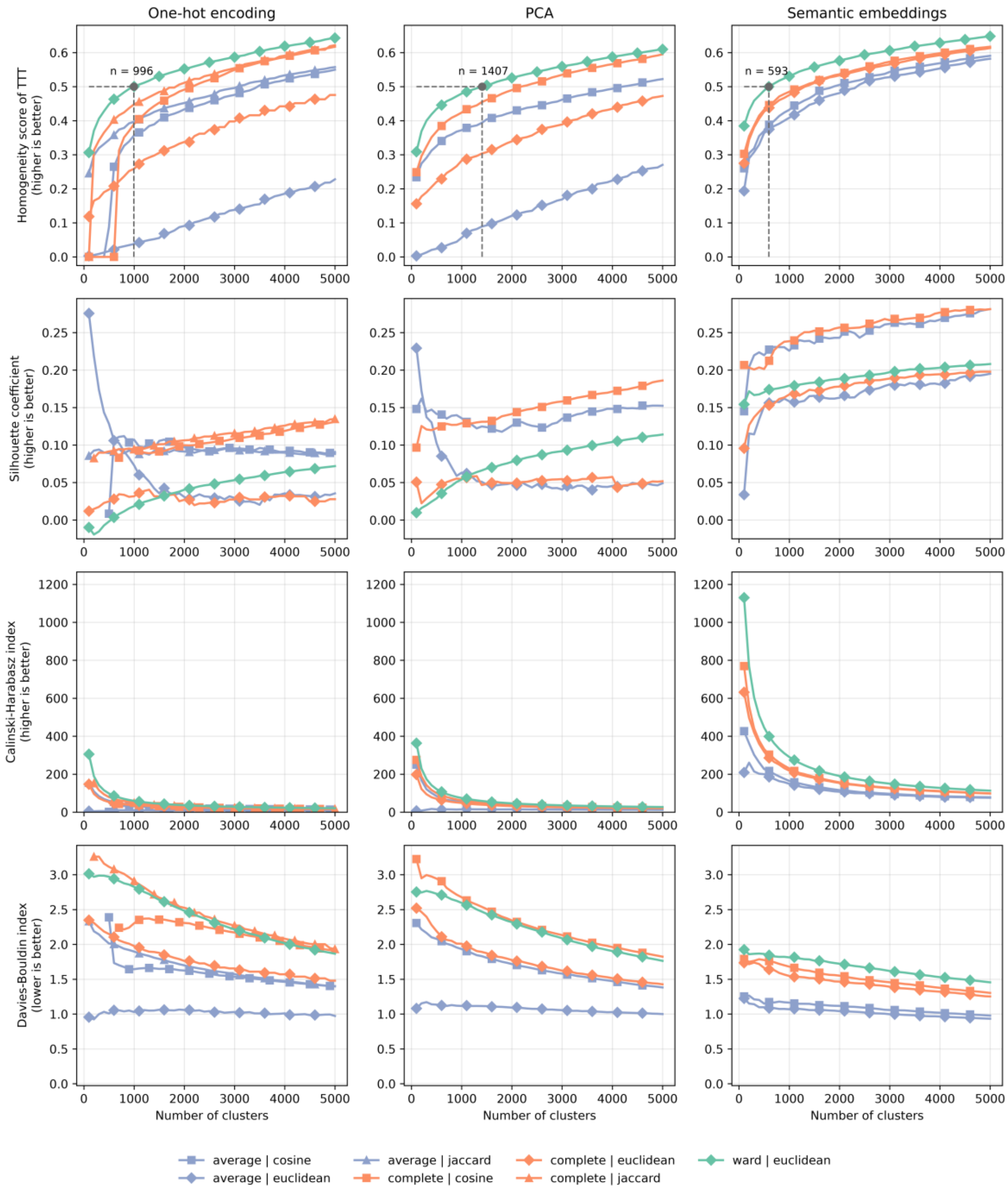

Figure S1: Agglomerative clustering performance: TTT homogeneity, silhouette coefficient, Calinski-Harabasz index, and Davies-Bouldin index are shown across numbers of clusters for all eligible agglomerative clustering configurations. Triage assessments were represented using one-hot encoding, one-hot encoding followed by principal component analysis (PCA) retaining at least 90% of the variance, or semantic embeddings generated with the Sentence Transformer model "German Semantic STS V2". Evaluated linkage methods were "average", "complete", and

“ward”. Evaluated distance metrics were “euclidean”, ”cosine”, and "jaccard”. Jaccard distance was evaluated only for one-hot encoded data, and Ward linkage only with Euclidean distance. The Sentence Transformer embeddings combined with Ward linkage and Euclidean distance achieved a TTT homogeneity score of 0.5 with the fewest clusters (n=593) and were therefore selected for subsequent analysis.

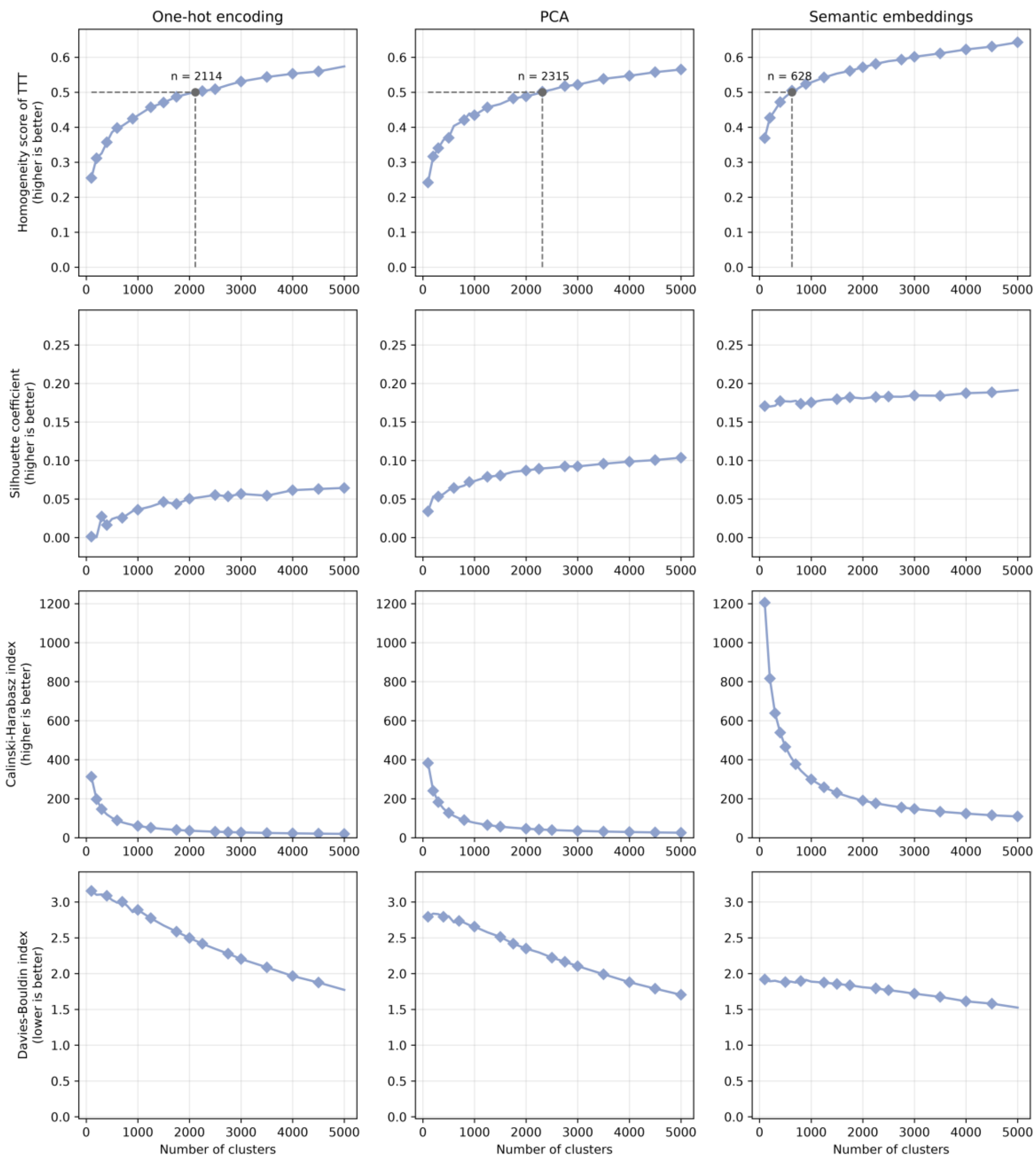

Figure S2: K-means clustering performance: TTT homogeneity, silhouette coefficient, Calinski-Harabasz index, and Davies-Bouldin index are shown across numbers of clusters for k-means clustering. Triage assessments were represented using one-hot encoding, one-hot encoding followed by principal component analysis

(PCA) retaining at least 90% of the variance, or semantic embeddings generated with the Sentence Transformer model "German Semantic STS V2". Because k-means is defined in Euclidean space, no alternative distance metrics were evaluated. Among the tested k-means configurations, semantic embeddings showed the most favorable performance, but a larger number of clusters was required to reach a TTT homogeneity of 0.5 than for agglomerative clustering (n=628 vs. n=593).

Table S1: HDBSCAN clustering performance across input representations and distance metrics. For each eligible HDBSCAN configuration, the number of identified clusters, the number and proportion of clustered assessments, TTT homogeneity, silhouette coefficient, Calinski-Harabasz index, and Davies-Bouldin index are shown. Triage assessments were represented using one-hot encoding, one-hot encoding followed by principal component analysis (PCA) retaining at least 90% of the variance, or semantic embeddings generated with the Sentence Transformer model "German Semantic STS V2". Evaluated distance metrics were "euclidean", "cosine", and "jaccard". Jaccard distance was evaluated only for one-hot encoded data. Because HDBSCAN may label low-density observations as noise, TTT homogeneity and internal validity indices were computed only for clustered assessments, while the number and proportion of clustered assessments are reported separately to quantify coverage. Configurations with higher TTT homogeneity generally clustered only a minority of assessments, whereas configurations with broader coverage showed lower TTT homogeneity.

Input	Distance metric	Cluster, n	Clustered assess-ments (N=50,000), n (%)	Homo-geneity of TTT	Silhouette coefficient	Calinski-Harabasz index	Davies-Bouldin index
One-hot encod-ing	cosine	219	21,308 (42.6)	0.40	0.01	52.72	7.96
	euclidean	297	40,472 (80.9)	0.22	-0.22	9.74	12.75
	jaccard	351	37,682 (75.4)	0.27	-0.04	26.00	11.27
PCA	cosine	80	12,651 (25.3)	0.52	0.53	261.68	1.23
	euclidean	57	5573 (11.1)	0.60	0.63	421.84	0.89
Semantic embedd-ings	cosine	118	22,036 (44.1)	0.47	0.52	1235.80	0.97
	euclidean	116	21,061 (42.1)	0.47	0.38	1296.80	0.95

Supplementary Results: Further Analyses of the Best-Performing Clustering Approach

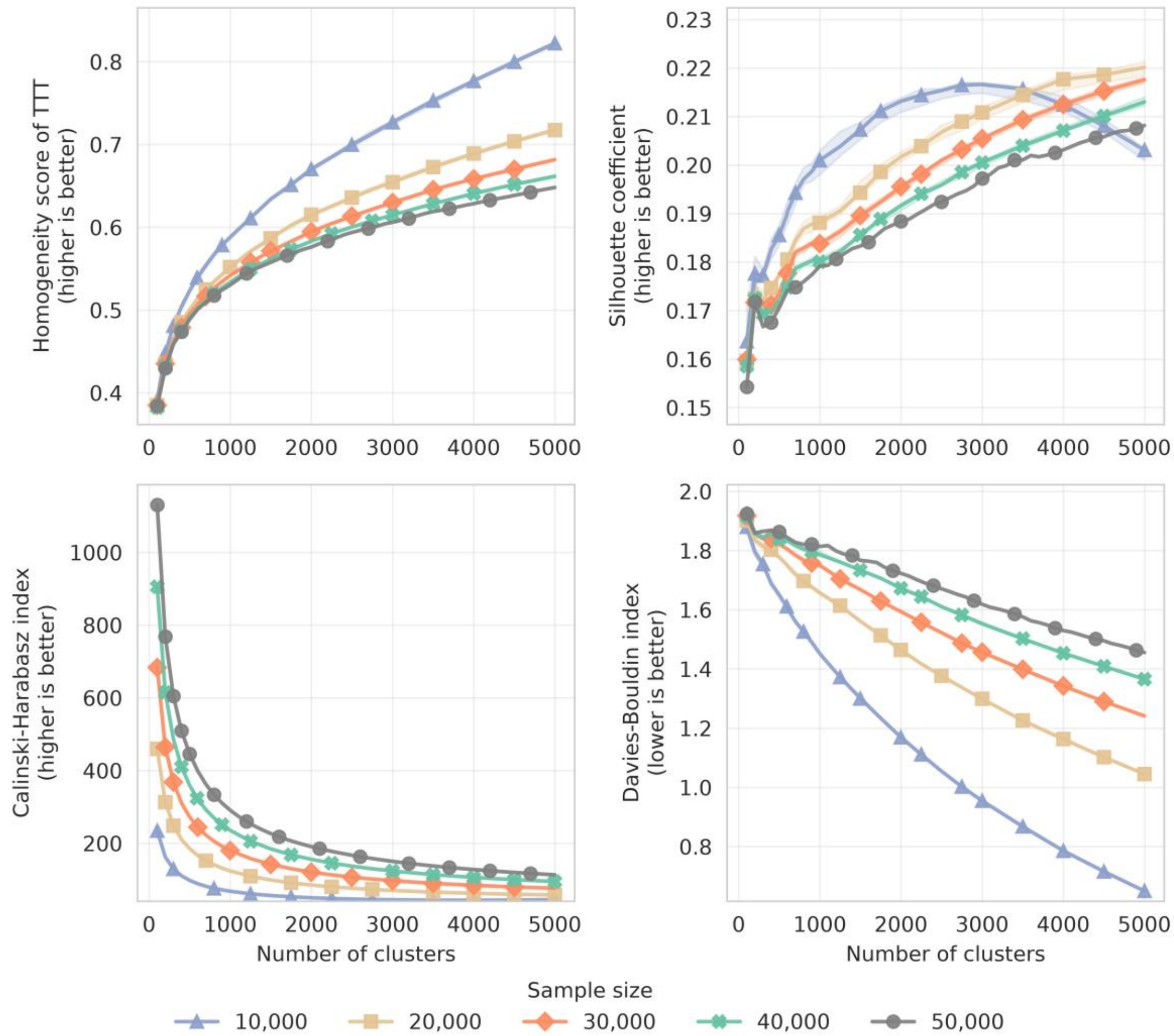

Figure S3: Sensitivity of agglomerative clustering performance by sample size: TTT homogeneity, silhouette coefficient, Calinski-Harabasz index, and Davies-Bouldin index are shown across numbers of clusters for the best-performing agglomerative clustering configuration (Sentence Transformer embeddings combined with Ward linkage and Euclidean distance). For each sample size (n=10,000, 20,000, 30,000, 40,000), ten subsamples were drawn from the original sample of 50,000 assessments. For each sample size and number of clusters, the median performance across the ten subsamples is shown together with the interquartile range, to assess robustness of clustering performance to sample size. Overall, clustering performance became less favorable with increasing sample size, but successive increases in sample size had progressively smaller effects.

Table S2: Sensitivity of cluster inclusion and covered assessments to the minimum cluster-size threshold. For the best-performing clustering configuration (agglomerative clustering with "German Semantic STS V2", Ward linkage and Euclidean distance), the number and proportion of clusters retained and sampled assessments covered are shown for different minimum cluster-size thresholds.

Minimum cluster size	Retained clusters (N=593), n (%)	Covered assessments (N=50,000), n (%)
30	498 (84.0)	48,143 (96.3)
40	433 (73.0)	45,892 (91.8)
50	377 (63.6)	43,431 (86.9)

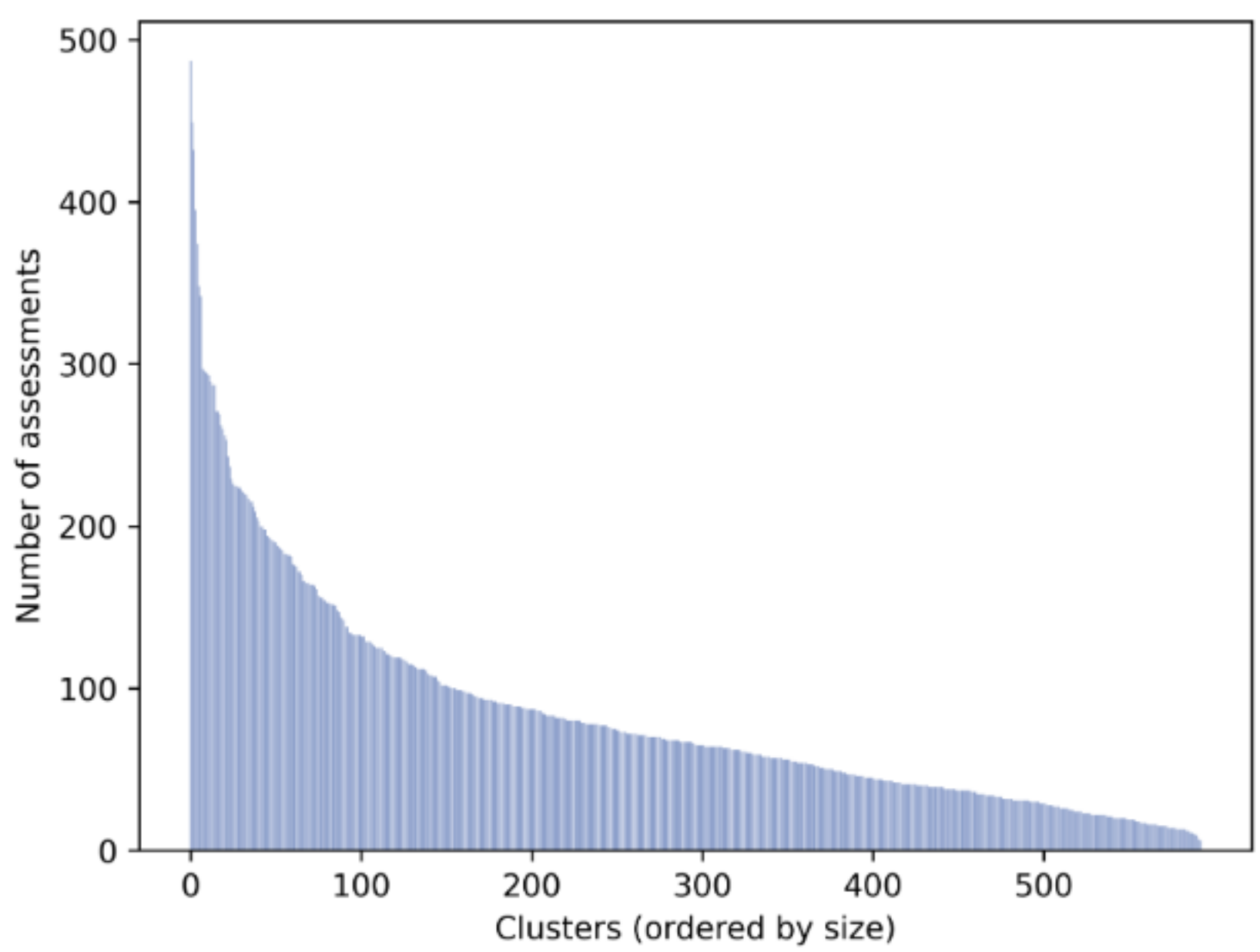

Figure S4: Number of triage assessments per cluster for the best-performing clustering configuration (agglomerative clustering with "German Semantic STS V2", Ward linkage and Euclidean distance). Clusters (n=593) were obtained by cutting the dendrogram at a threshold yielding a TTT homogeneity score of 0.5.

References

1. Rousseeuw PJ. Silhouettes: A graphical aid to the interpretation and validation of cluster analysis. *Journal of Computational and Applied Mathematics*. 1987;20:53-65. doi:10.1016/0377-0427(87)90125-7

2. Caliński T, Harabasz J. A dendrite method for cluster analysis. *Communications in Statistics*. 1974;3(1):1-27. doi:10.1080/03610927408827101

3. Davies DL, Bouldin DW. A Cluster Separation Measure. *IEEE Transactions on Pattern Analysis and Machine Intelligence*. 1979;PAMI-1(2):224-227. doi:10.1109/TPAMI.1979.4766909

4. Lukasová A. Hierarchical agglomerative clustering procedure. *Pattern Recognition*. 1979;11(5):365-381. doi:10.1016/0031-3203(79)90049-9

5. Ikotun AM, Ezugwu AE, Abualigah L, Abuhaija B, Heming J. K-means clustering algorithms: A comprehensive review, variants analysis, and advances in the era of big data. *Information Sciences*. 2023;622:178-210. doi:10.1016/j.ins.2022.11.139

6. McInnes L, Healy J, Astels S. hdbscan: Hierarchical density based clustering. *The Journal of Open Source Software*. 2017;2(11):205. doi:10.21105/joss.00205

7. Pearson K. LIII. On lines and planes of closest fit to systems of points in space. *The London, Edinburgh, and Dublin Philosophical Magazine and Journal of Science*. 1901;2(11):559-572. doi:10.1080/14786440109462720

8. aari1995/German_Semantic_STS_V2 · Hugging Face. Published online May 2024. Accessed May 27, 2026. https://huggingface.co/aari1995/German_Semantic_STS_V2

9. Virtanen P, Gommers R, Oliphant TE, et al. SciPy 1.0: Fundamental algorithms for scientific computing in Python. *Nature Methods*. 2020;17(3):261-272. doi:10.1038/s41592-019-0686-2

10. Pedregosa F, Pedregosa F, Varoquaux G, et al. Scikit-learn: Machine Learning in Python. *MACHINE LEARNING IN PYTHON*.
