# Supplementary material for: Generating Population-Linked Case Vignettes From Nationwide Triage Data: Framework Development and Validation Study
Source: JMIR Med Inform. 2026 Nov 3;14:e104801. doi: 10.2196/104801 (PMC13730294; doi:10.2196/104801)
Supplement: Supplementary file 2 [file medinform-v14-e104801-s002.pdf]

Multimedia Appendix

Additional methods and results for representative assessment generation and vignette-weight sensitivity analysis.

Supplementary Methods: Generation of Representative Assessments

Phase 1: Heuristic Search for Representative Cluster Assessments

For each cluster, we aimed to build one representative assessment x^* that best captures the cluster's key characteristics. First, the cluster centroid μ_C was computed as the mean of all 1024-dimensional sentence-embedding vectors of the assessments within the cluster. Next, the Euclidean distance between each assessment's sentence embedding $E(x)$ in the cluster and μ_C was calculated, and the assessment with the smallest distance to the centroid μ_C was selected as the initial representative assessment x_0. Subsequently, the empirical proposal distribution $P(e)$ was constructed from the frequency distribution of all question-answer combinations observed within the cluster, i.e. the proportion of assessments in which each question-answer state occurred. For each question occurring in the cluster, we additionally introduced an explicit "unanswered" state with probability corresponding to the proportion of cluster assessments in which that question has not been answered. Starting from x_0 and based on this frequency distribution, the initial assessment was iteratively refined using the general simulated annealing framework (see Textbox S1 below). We used simulated annealing because the search space is discrete and constrained by multiple questionnaire-consistency rules, so improving the final representative may require intermediate changes that temporarily worsen the current solution x. A purely greedy algorithm would therefore be more prone to becoming trapped in local minima. In addition, simulated annealing is a simple and well-established stochastic optimization approach for constrained discrete search problems and was straightforward to implement within our rule-based questionnaire framework.

A sampled edit $e \sim P(e)$ could replace the currently selected answer to a question, add an answer to a previously unanswered question, or remove an answer by setting the respective question to the "unanswered" state. After a proposed edit e was applied to the previous solution x to obtain x', rule-based constraints R were enforced to preserve questionnaire consistency (see Section "Consistency rules" below). Candidate assessments x' were evaluated using the objective function

$$f_{\text{Phase 1}}(x') = \text{distance}(E(x'), \mu_C)$$

where *distance* denotes the Euclidean distance. Simulated annealing was run for up to 10,000 iterations ($I_{\max}$), starting with an initial temperature T_0 of 1000, a minimum temperature $T_{\min}$ of 0.0001, and cooling factor α of 0.95. The generated assessment x^*achieving the smallest value of $f_{\text{Phase 1}}$ over all iterations was selected as the representative assessment for that cluster.

Textbox S1: Pseudocode of the simulated annealing framework for representative assessment generation.

```
Input: Initial assessment x_0; empirical proposal
distribution P(e); consistency rules R; objective function
f(x); maximum iterations I_max; initial temperature T_0;
minimum temperature T_min; cooling factor α
Output: optimized representative assessment x*, best
objective value c*
Initialize:
x ← x_0        // current assessment
c ← f(x)       // current objective value
T ← T_0        // current temperature
I ← 0          // current iteration
x* ← x         // best assessment found so far
c* ← c         // best objective value found so far
while T > T_min and I < I_max:
      I ← I + 1
      Sample candidate edit e ~ P(e)
      Apply e to x to obtain candidate assessment x′
      Enforce consistency rules R on x′
      if x′ is invalid or unchanged: continue
      Compute candidate objective value c′ ← f(x′)
      Compute Δ ← c′ − c
      Sample u ~ uniform(0,1)
      if Δ < 0 or u < exp(−Δ/T):
            // Accept candidate
            x ← x′
            c ← c′
            // Update best candidate
            if c < c*:
                  x* ← x
                  c* ← c
      T ← α * T // Update temperature
Return x*, c*
```

Phase 2: Enrichment of Representative Cluster Assessments

The representative assessments x^*obtained in Phase 1 captured key features of each cluster but can lack details regarding the reported symptoms. Therefore, we performed an additional enrichment step aimed at increasing the information density and realism of each representative assessment x^* while preserving the underlying symptom patterns identified in Phase 1. In Phase 2, the initial assessment x_0 was set to the Phase 1 representative assessment x^*. For each selected symptom in x_0, a relative importance weight w_{symp} was calculated by

removing all information related to that symptom from x_0, recomputing the assessment embedding $E(x_0)$, and recalculating the distance to the cluster centroid μ_C:

$$w_{\text{symp}} = \frac{distance\left(E\left(x_{0_{\text{w/o symptom}}}\right), \mu_C\right)}{\sum_{symp} distance\left(E\left(x_{0_{\text{w/o symptom}}}\right), \mu_C\right)}$$

Removing important symptoms increased the distance to the centroid more than removing less important ones, thereby obtaining a higher value for w_{symp}.

In Phase 2, the Phase 1 symptom set was kept fixed, and only symptom-specific detail questions (e.g., duration, severity, location) belonging to already selected symptoms were allowed to change. Accordingly, the empirical proposal distribution $P(e)$ was redefined to sample only from the cluster-specific distribution of symptom-specific *detail* questions belonging to already selected symptoms in x_0, again including an explicit "unanswered" state. After each proposed edit e, questionnaire consistency rules R were enforced (where applicable) to obtain candidate assessment x'. This assessment was evaluated using an objective function, which balanced proximity to the cluster centroid μ_C against the number of answered symptom-related questions, weighted by symptom importance:

$$f_{\text{Phase2}}(x') = \lambda \cdot \text{distance}(\text{E}(x'), \mu_C) - (1 - \lambda) \cdot \sum_{\text{symp}} \text{w}_{\text{symp}} \cdot \frac{\#\text{q}_{\text{symp}}(x')}{\#\text{q}_{\text{symp}}}$$

Here, $\#q_{\text{symp}}(x')$ denotes the number of symptom-specific detail questions answered for symptom *symp* in the current generated representative assessment x', and $\#q_{\text{symp}}$ denotes the number of such questions that were observed with an answer in the source cluster for that symptom. The weighting factor λ in [0,1] controls the trade-off between minimizing the distance to the centroid and maximizing the number of answered symptom-related questions. We selected λ=0.25 to favor more complete triage questionnaires while maintaining proximity to the cluster centroid. In a sensitivity analysis comparing λ=0.25 and λ=0.50, differences in centroid distance were small, whereas λ=0.25 yielded slightly richer representative assessments (see Table S1 below).

The simulated annealing framework as in Phase 1 (see Textbox S1) was then re-run applying the same parameter settings, but using the Phase 2-specific initialization x_0, proposal distribution $P(e)$, and objective function $f_{\text{Phase 2}}$. The generated assessment x^* with the smallest value of $f_{\text{Phase 2}}$ was retained as the final representative assessment for that cluster.

Consistency Rules

After each proposed edit e, rule-based constraints R were enforced to preserve questionnaire consistency:

- When a symptom was removed by setting a symptom-presence question to "no" or "unanswered", all associated symptom-specific detail questions were removed as well (set to "unanswered").
- Conversely, when a symptom-specific detail question was answered, the corresponding symptom-presence question was automatically set to "yes" if not already present.
- Symptom combinations were restricted to those permitted by the age- and gender-specific symptom structure of the triage system, and changes in age or gender triggered re-evaluation of symptom validity.
- Pregnancy-related questions were only allowed for female patients in the reproductive age range.
- Dependent child questions were removed whenever their required parent question-answer condition was no longer fulfilled.

Supplementary Results: Generation of Representative Assessments

Table S1: Sensitivity of the trade-off parameter λ used to balance cluster centrality with symptom-detail in the representative assessment generation Phase 2. A value of λ=0.5 weights cluster-proximity and answered questions equally, while λ=0.25 favors more answered triage questions.

λ	Representative assessments of Phase1 eligible for Phase 2 enrichment, n (%)	Representative assessments with detail enrichment after Phase 2, n (%)	Distance to the cluster centroid across all final representatives (N=514), mean, median (IQR)	Answered triage questions across all final representatives (N=514), mean, median (IQR)
0.25	458 (89.1)	289 (56.2)	2.1, 2.0 (1.6-2.5)	19.4, 17.0 (12.0-27.0)
0.50	458 (89.1)	212 (41.2)	2.0, 1.9 (1.5-2.3)	18.7, 17.0 (11.0-26.0)

Supplementary Methods: Sensitivity Analysis of Vignette Weights

To assess, whether cluster-based vignette weights were transferable to the full 1-year population, we conducted a similarity-based sensitivity analysis using semantic embeddings of representative and population assessments.

Concretely, each assessment from the generated vignette set and each assessment from the 1-year population was represented as a text document by concatenating the question-answer pairs and prefixing symptom-specific entries with the corresponding symptom name, analogous to the procedure used in the cluster analysis. These documents were embedded into a 1024-dimensional vector using the "German Semantic STS V2" Sentence Transformer Model. We then computed the pairwise cosine similarity between each vignette assessment and all assessments in the 1-year population. To determine an appropriate similarity threshold, we performed a calibration analysis based on symptom overlap. For similarity thresholds between 0.90 and 1.00, we first identified all vignette-population pairs exceeding the respective threshold and then calculated the mean Jaccard index between their sets of recorded symptoms, defined over the 121 symptom labels defined in the triage system. Under the assumption that recorded symptoms of semantically similar assessments should at least moderately overlap, we selected the threshold at which the mean Jaccard index reached a value of 0.5, resulting in a similarity threshold of 0.955.

Using this approach, each population assessment could be matched to multiple vignettes from the same set. To avoid double counting, each matched population assessment was assigned a fractional weight w_{ass} inversely proportional to the number of similar matched vignettes:

$$\mathrm{w}_{\text{ass}} = \frac{1}{\#\text{similar\_vignettes}}$$

The similarity-based weight per vignette w_{vignette} was then defined as the sum of the fractional weights of all similarly matched population assessments:

$$\mathrm{w}_{\text{vignette}} = \sum_{\text{ass} \in \text{similar\_assessments}_{\text{vignette}}} \mathrm{w}_{\text{ass}}$$

By construction, the sum of the similarity-based weights per vignette equals the total number of population assessments matched to at least one vignette above the selected similarity threshold. These similarity-based weights provide an alternative population-level estimate of vignette weight and were compared with the original cluster-based weights using Spearman rank correlation.
