# Supplementary material for: Generating Population-Linked Case Vignettes From Nationwide Triage Data: Framework Development and Validation Study
Source: JMIR Med Inform. 2026 Nov 3;14:e104801. doi: 10.2196/104801 (PMC13730294; doi:10.2196/104801)
Supplement: Supplementary file 3 [file medinform-v14-e104801-s003.pdf]

Multimedia Appendix

Mapping of triage symptoms to symptom categories.

Table S1: Mapping of 121 symptoms recorded by the triage system (translated from German) to 13 broader symptom categories. Three symptoms could not be assigned to a specific symptom category due to their general nature and are therefore listed as “N/A” in the symptom category column.

**Symptom category**	**Symptom**
Cardiovascular system	Lymph node problems
Cardiovascular system	Blood pressure problem
Cardiovascular system	Chest pain
Cardiovascular system	Heart palpitations
Cardiovascular system	Heart rhythm disorders
Cardiovascular system	Fainting
Digestive system	Jaundice
Digestive system	Rectal bleeding
Digestive system	Anal pain
Digestive system	Stomach pain
Digestive system	Diarrhea
Digestive system	Vomiting/nausea
Digestive system	Swallowed a foreign object
Digestive system	Mouth, tongue, or lip problems
Digestive system	Black stool/Blood in the stool
Digestive system	Lower abdominal pain
Digestive system	Indigestion
Digestive system	Constipation
Digestive system	Tooth/gum problems
Digestive system	Groin pain
Ears	Hearing problems
Ears	Foreign object in the ear
Ears	Ear discharge
Ears	Earache
Ears	Ringing in the ears / Tinnitus
Eyes	Eye redness
Eyes	Eye pain
Eyes	Vision problems
Infectious/parasitic	Fever
Infectious/parasitic	Pubic lice
Infectious/parasitic	Head lice
Injuries/poisonings/external causes	Vaccine reaction
Injuries/poisonings/external causes	Arm injury (due to an accident)

Injuries/poisonings/external causes	Eye injury
Injuries/poisonings/external causes	Abdominal injury
Injuries/poisonings/external causes	Leg injury (due to an accident)
Injuries/poisonings/external causes	Chest injury
Injuries/poisonings/external causes	Inhalation foreign object/toxic substance
Injuries/poisonings/external causes	Elbow injury (due to an accident)
Injuries/poisonings/external causes	Finger injury (due to an accident)
Injuries/poisonings/external causes	Foot injury (due to an accident)
Injuries/poisonings/external causes	Facial injury
Injuries/poisonings/external causes	Plaster-related issues
Injuries/poisonings/external causes	Wrist injury (due to an accident)
Injuries/poisonings/external causes	Hand injury (due to an accident)
Injuries/poisonings/external causes	Hip injury (due to an accident)
Injuries/poisonings/external causes	Insect sting/bite
Injuries/poisonings/external causes	Knee injury (due to an accident)
Injuries/poisonings/external causes	Head injury
Injuries/poisonings/external causes	Nose injury
Injuries/poisonings/external causes	Back injury
Injuries/poisonings/external causes	Shoulder injury (due to an accident)
Injuries/poisonings/external causes	Sunstroke / Heat-related illnesses
Injuries/poisonings/external causes	Ankle injury (due to an accident)
Injuries/poisonings/external causes	Electrical accident / electric shock
Injuries/poisonings/external causes	Accident/Trauma
Injuries/poisonings/external causes	Burn/Scald
Injuries/poisonings/external causes	Poisoning
Injuries/poisonings/external causes	Wound/skin injury
Injuries/poisonings/external causes	Toe injury (due to an accident)
Mental health	Anxiety/restlessness/tension
Mental health	Depressive feeling
Mental health	Panic attack
Mental health	Sleep disorders
Mental health	Suicidal thoughts/Suicidal tendencies
Mental health	Strange thoughts
Mental health	Strange behavior
Metabolic	Loss of appetite
Metabolic	Blood sugar problem
Metabolic	Change in weight
Musculoskeletal system	Leg problems
Musculoskeletal system	Elbow pain
Musculoskeletal system	Finger problems
Musculoskeletal system	Foot pain

Musculoskeletal system	Joint pain
Musculoskeletal system	Hand problems
Musculoskeletal system	Wrist pain
Musculoskeletal system	Hip pain
Musculoskeletal system	Knee problems
Musculoskeletal system	Back/Thoracic Spine Pain
Musculoskeletal system	Lumbar back pain
Musculoskeletal system	Cervical back pain
Musculoskeletal system	Shoulder pain
Musculoskeletal system	Ankle pain
Musculoskeletal system	Toe problems
Musculoskeletal system	Arm pain
Nervous system	Headache
Nervous system	Neurological seizure
Nervous system	Dizziness
Nervous system	Speech disorder
Nervous system	Facial pain
Nervous system	Muscle weakness
Respiratory system	Asthma symptoms
Respiratory system	Breathing problems
Respiratory system	Cold/influenza infection
Respiratory system	Suspected COVID-19
Respiratory system	Throat/pharyngeal pain
Respiratory system	Cough
Respiratory system	Nosebleed
Respiratory system	Foreign object in the nose
Respiratory system	Difficulty swallowing
Urogenital	Penile discharge
Urogenital	Vaginal discharge
Urogenital	Gynecological symptoms
Urogenital	Genital discomfort in men
Urogenital	Blood in semen
Urogenital	Urinary tract problems
Urogenital	Pain in the testicles or scrotum
Urogenital	Vaginal bleeding
Urogenital	Foreskin problem
Urogenital	Breast problem
Skin	Abscess
Skin	Allergic symptoms
Skin	Rash
Skin	Dark spot on the skin

Skin	Itching
Skin	Lump/Swelling
Skin	Sunburn
N/A	Fatigue
N/A	Feeling unwell
N/A	Crying baby/toddler
