# Supplementary material for: Generating Population-Linked Case Vignettes From Nationwide Triage Data: Framework Development and Validation Study
Source: JMIR Med Inform. 2026 Nov 3;14:e104801. doi: 10.2196/104801 (PMC13730294; doi:10.2196/104801)
Supplement: Supplementary file 4 [file medinform-v14-e104801-s004.pdf]

Multimedia Appendix

Supplementary methods and results of the gender-effects sensitivity analysis.

Supplementary Methods: Gender-Effects Sensitivity Analysis

For the gender-effects sensitivity analysis, representative assessments were first classified as a priori gender-specific if they contained questionnaire content that was inherently specific to one gender.

Representative assessments were classified as female-specific if they contained at least one of the following female-specific symptom labels: “Vaginal discharge”, “Gynecological symptoms, “Breast problem”, or “Vaginal bleeding”, or if they contained answered questions related to pregnancy, postpartum status or breastfeeding, menstruation, menopause, menstrual bleeding, or menstrual cycle.

Representative assessments were classified as male-specific if they contained at least one of the following male-specific symptom labels: “Penile discharge”, “Genital discomfort in men”, “Blood in semen”, “Pain in the testicles or scrotum”, or “Foreskin problem”, or if they contained answered questions referring to testicles or penis.

All representative assessments not fulfilling any of these criteria were classified as non-gender-specific. For each non-gender-specific representative assessment, a gender-modified version was created by changing the recorded gender to male or female while preserving all other question-answer pairs. Each modified assessment was then re-submitted to the triage system, and the resulting TTT and POC recommendations were compared with those of the original representative assessment.

Supplementary Results: Gender-Effects Sensitivity Analysis

Among the 514 final representative assessments, 23 were classified as a priori gender-specific, including 19 female-specific and 4 male-specific representatives. The remaining 491 representative assessments were classified as non-gender-specific and were included in the gender-effects sensitivity analysis. Changing the recorded gender altered the resulting TTT and/or POC recommendation in 3 of the 491 non-gender-specific representative assessments. All three cases concerned urinary-tract complaints and resulted in higher urgency in the male version. Based on the original cluster-based weights, these three representative assessments corresponded to a total of 306 underlying assessments.
