# Supplementary material for: Generating Population-Linked Case Vignettes From Nationwide Triage Data: Framework Development and Validation Study
Source: JMIR Med Inform. 2026 Nov 3;14:e104801. doi: 10.2196/104801 (PMC13730294; doi:10.2196/104801)
Supplement: Supplementary file 5 [file medinform-v14-e104801-s005.pdf]

Multimedia Appendix

Supplementary methods and results regarding case vignette generation.

Supplementary Methods: Case Vignette Generation

After identifying one representative assessment per cluster, we aimed to formulate a specific patient vignette for each case using an LLM. We used GPT-4.1 (OpenAI, Generative Pre-Trained Transformers) and prompted it in German (because the triage questions were originally in German and we wanted to avoid language mixing) to generate a case vignette based on the question-answer pairs of each representative assessment. The model was instructed to:

- Use *only* the information contained in the given questions and answers and avoid adding assumptions or external knowledge (to prevent hallucinations).
- Include *all* answered questions in the vignette, regardless of apparent relevance (to prevent information loss).
- Structure the vignette in three sections: 1. Patient information (age, gender, pregnancy, if applicable), 2. Medical history (structured by symptoms or symptom complexes), 3. Other medically relevant information (e.g., risk factors, medications, pre-existing conditions, lifestyle)
- Use a medically precise, factual style of language.
- Select a specific value for interval expressions by sampling from a uniform distribution for closed intervals (e.g., age between 9 and 13 years, pain intensity between 4 and 7), and from an asymmetric distribution for open intervals (e.g., more than 3 days, older than 80 years, body temperature > 30 °C, blood pressure < 90 mmHg) assigning higher probabilities closer to values near the stated boundary.

This structure was chosen to standardize the presentation of the vignettes and to facilitate rapid orientation and systematic review by medical experts by separating patient descriptors, symptom-related history, and other clinically relevant contextual information. A medically precise, factual style was preferred over user-oriented language because the primary aim of the vignettes was expert evaluation and benchmarking rather than simulation of lay patient speech. Although user-oriented language might have increased narrative naturalism, it would also have introduced additional stylistic variability not grounded in the source data.

The final instruction, selecting precise values within intervals, was necessary because the representative assessments were derived from triage data, and the triage system often records information in broad categories rather than exact values (for instance, duration of symptoms, age, pain intensity, or vital signs). To simulate

realistic patient cases, these categorical ranges had to be converted into specific values. While the LLM handled most interval expressions correctly, it occasionally retained interval expressions for vital signs (e.g., blood pressure between 120 and 140 mmHg). Therefore, we added a preprocessing step that algorithmically assigned precise numerical values for vital-sign-related questions. All triage questions capturing vital signs were identified through algorithmic analysis of their possible answers, and each affected representative assessment was assigned a random value within the corresponding range. Closed intervals were sampled from a uniform distribution, whereas open intervals were drawn from an exponential distribution within physiologically plausible limits for human vital signs. The complete prompt is provided in Textbox S1 below for transparency and reproducibility.

Textbox S1: LLM system prompt (translated from German) to generate case vignettes from triage questionnaires.

You will receive information about a patient in a question-and-answer format.
Please note: Questions preceded by a symptom name refer exclusively to that specific symptom.
Your task is to write a patient vignette (case description) solely on the basis of this information.
Use all given questions and answers, including negations or information that you may consider irrelevant.
Under no circumstances should you add your own assumptions.
Structure the case vignette as follows:
1. Patient: age, sex, and, if applicable, pregnancy.
2. Medical history (anamnesis): Structure this section according to the different symptoms. Each symptom should form a short paragraph. If appropriate in terms of content, symptoms may also be grouped into symptom complexes.
3. Further medically relevant information: This section should include information on risk factors, medications, pre-existing conditions, lifestyle, etc., if these have not already been mentioned in the medical history.
If no information is available for a section, write "No information provided" under the respective heading.
Write in complete sentences in a medically precise, factual, and clear style.
Do not make any diagnoses. Do not add any additional information - write solely based on the questions and answers provided.
If an answer contains an interval, choose a specific value from that interval according to the following rules:
- For closed intervals (e.g., "between 10 and 20 years"), choose a representative value from the interval using a uniform distribution.
Examples: "Age between 10 and 20 years": e.g., "14 years"; "Duration 2 to 3 days": e.g., "3 days"; "Moderate pain (4–7 out of 10)": e.g., "moderate pain (5 out of 10)"; "Vomiting 1–3 times": e.g., "vomited twice."
- For open intervals (e.g., "older than 80 years"), use an asymmetric probability distribution in which values close to the interval boundary are more likely than those farther away:
- For expressions such as "greater than X," use an exponential distribution that starts at X and declines exponentially.
Example: "older than 80 years": more likely: 81 years; less likely: 98 years.
- For expressions such as "less than X," use a mirrored exponential distribution so that values close to X are more likely.
Example: "For less than one month": more likely: 28 days; less likely: 3 days.
Try to avoid double negations or statements that can only be inferred indirectly, and instead formulate them as clearly understandable affirmative statements. For example, "No bloating for a period of more than 48 hours" becomes "There has been no persistent bloating lasting more than 48 hours.", and "Does the bleeding continue despite pressure being applied for at least 15 minutes? Answer: No" becomes "There is no prolonged bleeding lasting more than 15 minutes."
Now create the case vignette for the following questions and answers:

Supplementary Results: Exemplary Case Vignettes and Quality Analysis

Data S1: Selection of five exemplary case vignettes (translated from German).

1. Patient: The patient is a 61-year-old man.
2. Medical history (anamnesis): Leg complaints: The patient reports bilateral leg complaints that have been present for 4 days. The complaints are described as severe (8 out of 10). There is newly developed swelling of both legs. There are no indications of inflammation such as warmth or redness. Signs of circulatory disturbance were also denied. Weight-bearing on the legs is currently not possible.
3. Further medically relevant information: There are no known risk factors for the development of blood clots (thrombosis or embolism) in the leg, arm, or lung. No further information is available.

---

1. Patient: The patient is an 81-year-old man.
2. Medical history (anamnesis): Abdominal pain and digestive disorder: The patient complains since today of severe, cramping or colicky abdominal pain that is poorly localized and radiates to the back. In addition, he reports a digestive disorder. The pain in the context of the digestive disorder is localized in the middle upper abdomen and is perceived as painful. Furthermore, the patient suffers from heartburn or acid regurgitation. There is flatulence, but not prolonged (>48 hours), and intestinal gas is still being passed. A recently performed abdominal operation as well as a stomach or intestinal endoscopy have not taken place in the past few days. The patient reports no ingestion of spoiled food.
3. Further medically relevant information: The patient has so far undertaken self-treatment measures using home remedies or over-the-counter medications. No relevant previous illnesses, specific risk factors, or known underlying diseases are reported.

---

1. Patient: The patient is a 63-year-old woman.
2. Medical history (anamnesis): Symptom of eye pain: Moderate eye pain (5 out of 10) has been present for 3 days. A specific eye was not mentioned. A small, painful, reddened, pus-filled nodule at the eyelid margin (stye) is present. The eyelids are reddened and swollen, but the eye can be opened. There are no stuck-together eyes or visible pus on the eyes. There are no indications of a foreign body in the eye, prior eye surgeries within the last 14 days, known eye diseases, or recent intensive light exposure (for example at the sea, in snow, due to electric welding or laser). No measures of self-treatment, such as home remedies or over-the-counter medications, have been taken.
3. Further medically relevant information: The patient wears contact lenses. There is no acutely life-threatening condition.

1. Patient: Female, 81 years old<br>2. Medical history (anamnesis): Fall event: The patient fell today. The fall occurred from a height lower than her own body height. There was no loss of consciousness. After the fall, walking and standing remain possible. There are no indications of movement disorders or skin sensory disturbances such as numbness or tingling.<br>3. Other medically relevant information: The patient is taking anticoagulant medication or has a known blood coagulation disorder. There is no immediately life-threatening situation.

1. Patient: The patient is a 68-year-old female.<br>2. Medical history (anamnesis): Cardiovascular complaints: The patient reports newly occurred cardiovascular symptoms. Specifically, she presents with a pale, sallow facial color and is cold-sweaty. She also reports recently developed severe cardiovascular complaints. The patient is breathing adequately and shows a normal response to verbal stimuli.<br>3. Other medically relevant information: No information provided.

Table S1: Findings from a manual review comparing 50 randomly selected LLM-generated case vignettes with their underlying representative assessments, comprising 761 question-answer pairs in total.

**Error category**	**Source information in representative assessment (summary or question-answer pair)**	**Content in vignette or explanation**	**n**
**Hallucination**			
	A fall resulting in moderately severe leg complaints, including swelling.	Vignette with judgment: “The fall resulted in injuries that require medical evaluation.”	1
**Misrepresented information**			
	Breathing difficulties: How long have the breathing difficulties been present? 2–7 days; How quickly did the symptoms appear? Unclear	Vignette: “The patient has reported mild shortness of breath (1 out of 10) for the past 4 days, [...]. The exact time when the symptoms began is unclear.”	1
	Have you been experiencing bloating for more than 48 hours and/or are you no longer	Vignette: “There is bloating, but it has not lasted for 48 hours yet.”	1

	passing gas (no more farts)? Bloating		
	Have you recently experienced a major life event, such as the loss of a loved one, a divorce, or a separation? Yes	Vignette: "No major life events, such as the loss of a loved one, divorce, or separation, have been reported."	1
	Is the person taking any medications that could damage the stomach lining? Unclear	Vignette: "No medications that could damage the stomach lining are being taken, or it is unclear whether this is the case."	1
**Missing information**			
	Is there an acute life-threatening situation? No	The LLM was instructed not to make any assumptions, assessments, or diagnoses. This conflicts with the triage system's preliminary triage question regarding whether a life-threatening case is present. This information was removed from all vignettes during medical expert review because it was considered to constitute an unintended assessment of the patient's condition.	19
	Is there an acute life-threatening situation? Unclear		2
	Fever: How long has the fever been going on? Unclear	Missing from the vignette	1
	Headaches: Did the headache occur while performing a strenuous activity? No	Missing from the vignette	1
	Headaches: How did the pain start? Unclear	Missing from the vignette	1
